# Dietary Supplementation With *Lactobacillus plantarum* Ameliorates Compromise of Growth Performance by Modulating Short-Chain Fatty Acids and Intestinal Dysbiosis in Broilers Under *Clostridium perfringens* Challenge

**DOI:** 10.3389/fnut.2021.706148

**Published:** 2021-10-14

**Authors:** Baikui Wang, Yuanhao Zhou, Yulong Mao, Li Gong, Xiang Li, Shujie Xu, Fei Wang, Qianpeng Guo, Huihua Zhang, Weifen Li

**Affiliations:** ^1^Key Laboratory of Molecular Animal Nutrition of the Ministry of Education, Key Laboratory of Animal Nutrition and Feed Science (Eastern of China) of the Ministry of Agriculture, Key Laboratory of Animal Feed and Nutrition of Zhejiang, College of Animal Sciences, Institute of Animal Nutrition and Feed Sciences, Zhejiang University, Hangzhou, China; ^2^School of Life Science and Engineering, Foshan University, Foshan, China

**Keywords:** *Lactobacillus plantarum*, *Paenibacillus polymyxa*, *Clostridium perfringens*, necrotic enteritis, growth performance, short-chain fatty acids, intestinal health, intestinal dysbiosis

## Abstract

*Clostridium perfringens* is an important zoonotic pathogen associated with food contamination and poisoning, gas gangrene, necrotizing enterocolitis or necrotic enteritis in humans and animals. Dysbacteriosis is supposedly associated with the development of *C. perfringens* infection induced necrotic enteritis, but the detailed relationship between intestinal health, microbiome, and *C. perfringens* infection-induced necrotic enteritis remains poorly understood. This research investigated the effect of probiotics on the growth performance and intestinal health of broilers, and the involved roles of intestinal microbiota and microbial metabolic functions under *C. perfringens* infection. Results showed that subclinical necrotic enteritis was successfully induced as evidenced by the significant lower body weight (BW), suppressed feed conversion ratio (FCR), decreased ileal villus height and mucosal barrier function, and increased ileal histopathological score and bursal weight index. *Lactobacillus plantarum* or *Paenibacillus polymyxa* significantly attenuated *C. perfringens*-induced compromise of growth performance (BW, FCR) and ileal mucosa damage as illustrated by the increased ileal villus height and villus/crypt ratio, the decreased ileal histopathological score and the enhanced ileal mucosal barrier function. *L. plantarum* also significantly alleviated *C. perfringens*-induced enlarged bursa of fabricius and the decreased levels of ileal total SCFAs, acetate, lactate, and butyrate. Furthermore, dietary *L. plantarum* improved *C. perfringens* infection-induced intestinal dysbiosis as evidenced by significantly enriched short-chain fatty acids-producing bacteria (*Lachnospiraceae, Ruminococcaceae, Oscillospira, Faecalibacterium, Blautia*), reduced drug-resistant bacteria (*Bacteroides, Alistipes*) and enteric pathogens (*Escherichia coli, Bacteroides fragilis*) and bacterial metabolic dysfunctions as illustrated by significantly increased bacterial fatty acid biosynthesis, decreased bacterial lipopolysaccharide biosynthesis, and antibiotic biosynthesis (streptomycin and vancomycin). Additionally, the BW and intestinal SCFAs were the principal factors affecting the bacterial communities and microbial metabolic functions. The above findings indicate that dietary with *L. plantarum* attenuates *C. perfringens*-induced compromise of growth performance and intestinal dysbiosis by increasing SCFAs and improving intestinal health in broilers.

## Introduction

*Clostridium perfringens* (*C. perfringens*) is a widely distributed anaerobic spore-forming zoonotic pathogen, which causes foodborne illnesses in humans and necrotic enteritis in animals (1, 2). Foodborne illness caused by *C. perfringens*-contaminated food in the United States is estimated to be nearly 1 million cases per year (2). Necrotic enteritis induced by *C. perfringens* is a widespread avian intestinal necrotic disease, which is estimated to cause the total global economic loss in poultry industry to be over US$6 billion annually (3). Clinical form of necrotic enteritis is characterized by sudden death and increased mortality of chickens, with a mortality rate from 2 to 50% (4). Subclinical infection leads to disrupted villus-crypt micro-architecture and reduced nutrient digestion and absorption, which adversely decreases feed conversion and impairs growth performance (4, 5). Infeed antibiotics used to be the main strategy for preventing or controlling necrotic enteritis in poultry production. However, with the increasing public concerns about antimicrobial resistance and antibiotic residues in food animal products, infeed antimicrobial growth promoters have been widely removed from the animal feed by increasing global countries (6, 7). Subsequently, outbreaks of necrotic enteritis have become a significant economic concern for poultry farmers, especially in subclinical form, which shows unobvious pathological symptoms and thereby compromises the growth performance (8). The withdrawal of infeed prophylactic antibiotics and outbreaks of necrotic enteritis in the commercial poultry industry inspires an interest in seeking effective alternative antimicrobial strategies to prevent or control necrotic enteritis outbreaks. In recent years, multiple dietary alternatives to prophylactic antibiotics, such as probiotics, prebiotics, plant extracts, enzymes, and organic acids, have been proved to be effective in reducing or abolishing *C. perfringens*-induced necrotic enteritis (5, 9).

Intestinal microbes play crucial roles in the development of the intestinal defense system (immune function and barrier function) and in regulating the processes of inflammation and maintaining homeostasis (10–12). It is reported that the anomalous intestinal microbiota is associated with the development of necrotic enteritis in animals or necrotizing enterocolitis in humans, but interactive roles of intestinal microbes in this relationship remain poorly understood (13, 14). As a potential advanced alternative to antibiotic growth promoters, many studies have shown that probiotics and commensal microbes exert beneficial effects on inhibiting growth and toxin secretion of pathogens, modulating gastrointestinal immune systems against adhesion and invasion of pathogens, restoring altered intestinal microbes, maintaining gastrointestinal homeostasis, and promoting tissue healing (15–17). Many studies have proved that *L. plantarum* exerts antibacterial activities by secreting lactic acid (18) and plantaricin (19, 20), and *P. polymyxa* exerts antibacterial activities by secreting polymyxin and lantibiotic (21, 22). Although numerous studies have demonstrated that probiotics, such as *lactobacillus, bacillus*, and yeast, could alleviate or abolish *C. perfringens* infection-induced necrotic enteritis (23–26), the involved interactive roles of intestinal microbiota, microbial metabolic functions, and short-chain fatty acids (SCFAs) under *C. perfringens* infection-induced necrotic enteritis remain poorly understood. Our previous works found that two probiotics, *Lactobacillus plantarum* (Lac16) and *Paenibacillus polymyxa* (BSC10), had *in vitro* anti-*C. perfringens* activities and Lac16 protected-*Caenorhabditis elegans* against *C. perfringens* infection (Supplementary Figure 1). The present study further evaluated the effect of the two probiotics on growth performance and intestinal health of broilers challenged with *C. perfringens*, and the interactive roles of intestinal microbial communities, bacterial metabolic functions, and SCFAs under *C. perfringens* infection condition.

## Experimental Procedures

### Bacteria Preparation

*L. plantarum* (Lac16) deposited in China Center for Type Culture Collection (CCTCC M2016259) was isolated from fermented vegetables, and *P. polymyxa* (BSC10) was purchased from China General Microbiological Culture Collection Center (CGMCC1.10711). The Lac16 and BSC10 were separately cultured in DeMan-Rogosa-Sharpe and Luria-Bertani broth at 37°C for overnight under aerobic conditions. *Clostridium perfringens* type A (ATCC13124, Cp) was cultured in reinforced clostridium medium at 37°C for 20 h in anaerobic gas-generating packs (Mitsubishi Gas Chemical Company Inc., Tokyo, Japan). After centrifugation at 3,500 × *g* for 10 min at 4°C, the BSC10, Lac16, and Cp pellets were collected and then washed three times with sterile phosphate-buffered saline (PBS, pH 7.2), respectively. Finally, the concentration of the bacteria was constantly checked by spreading the plate method (27).

### Chicken Experiment

Seven hundred and twenty hatched 1-day-old Cobb 500 broilers with similar body weight were randomly allocated into four treatments with six pens per group and 30 birds per pen: (1) Control group: birds were fed a basal diet (Supplementary Table 1) formulated to meet the nutritional requirements of broilers (28), (2) Cp group: birds were fed a basal diet and then orally challenged with *C. perfringens* type A cultures [1. × 10^8^ colony-forming units (CFU)/bird] on day 1 and during day 14–20 of age, (3) BSC10+Cp group: birds were fed a basal diet supplemented with *P. polymyxa* (1. × 10^8^ CFU/kg feed) and orally challenged with *C. perfringens* A cultures (1. × 10^8^ CFU/bird) on day 1 and during day 14–20 of age, (4) Lac16 + Cp group: birds were fed a basal diet supplemented with *L. plantarum* (1. × 10^8^ CFU/kg feed) and orally challenged with *C. perfringens* A cultures (1. × 10^8^ CFU/bird) on day 1 and during day 14–20 of age. The animal experiment lasted for 21 days. Birds were allowed *ad libitum* access to mashed diets and fresh water. Feed consumption and body weight were recorded every week. Mortality was recorded every day, and dead birds were weighed to adjust estimates of body weight gain, feed intake, and feed conversion ratio.

### Sample Collection

At day 21 of age, six birds from each group were randomly selected, weighed, and euthanized by electrical stun after deprivation of feed for 6 h (05:00–11:00 A.M.). After being removed and weighed, spleen and bursa of fabricius indexes were calculated as a percentage relative to body weight. The ileal segments were fixed in 4% paraformaldehyde for hematoxylin and eosin (H&E) staining or in 2.5% buffered glutaraldehyde for transmission electron microscopy (TEM). The ileal contents and whole caecum of birds were sampled, snap frozen in liquid nitrogen, and stored at −80°C for short-chain fatty acids (SCFAs) and microbial analysis.

### Ileal Morphological Analysis

After being fixed in 4% paraformaldehyde, ileal samples were embedded in paraffin, sliced, dehydrated, and stained with hematoxylin and eosin. Images were observed by Olympus microsystem (Tokyo, Japan). Histopathological scores of the ileal samples were examined by three independent observers as previously described (29).

For TEM observation, after being fixed in 2.5% buffered glutaraldehyde, ileal segments were washed three times by a cold 100-mM phosphate buffer, and then postfixed in cold 0.1% buffered osmium tetroxide (OsO_4_) for 2 h. After being washed by a phosphate buffer, the ileal segments were rapidly dehydrated in ascending grades of ethanol (30, 50, 70, 95, and 100%), and then transferred into a 1:1 mixture of propylene oxide and epoxy araldite. The ultrathin ileal sections were embedded and cut by an LKB Nova ultramicrotome (Leica Microsystems, Buffalo Grove, IL) and then stained with uranyl acetate. Transmission electron micrographs of the ileal samples were then observed and captured by the transmission electron microscope (JEOL, Tokyo, Japan).

### Lactate and SCFAs Analysis

Lactate levels in ileal digesta were determined by the Lactic Acid assay kit (NanJingJianCheng Bioengineering Institute, Nanjing, China) according to the instructions of the manufacturer. SCFAs contents in ileal digesta were measured as follows: 1 g of digesta was mixed with 2. ml of distilled water. After being homogenized and centrifuged at 5,000 × g for 10 min at 4°C, 500 μl of the supernatant were mixed with 0.2 ml 25% (w/v) phosphoric acid and then stored at −20°C for overnight. After thawing, the mixtures were centrifuged (15,000 × g for 10 min at 4°C), filtered by 0.22-μm membrane filter and then analyzed by gas chromatography (Varian CP-3800, USA). Quantification of SCFAs was carried out by using the external calibration standard curves method and expressed as μmol/g of wet ileal digesta.

### Microbial Analysis

The bacterial genomic DNA from cecal contents was extracted under sterile conditions using the TIANamp Stool DNA Kit (Tiangen, Beijing, China) and was stored at −80°C for PCR amplification and sequencing. The V3-V4 hypervariable region of the 16S rRNA gene was amplified by using the 341F/805R primer pairs, and the amplicon sequencing was performed on an Illumina MiSeq platform (Illumina Inc., San Diego, CA, USA). The Quantitative Insights into Microbial Ecology (QIIME) software (version 1.9.1) was used for the quality filter of raw sequences and a cluster of filtered sequences into operational taxonomic unit (OTU) at 97% similarity (30). Bacterial OTU representative sequences were assigned to a taxonomic lineage by a Ribosomal Database Project (RDP) classifier based on the Greengenes 13.8 database.

Alpha and beta diversities of the microbial community were analyzed based on a subsample of a minimum number of sequences (12981) by QIIME software. Significant differences in microbial beta diversity and metagenome predicational functions among different groups (based on the Bray-Curtis distance matrices) were calculated by ANOSIM (analysis of similarities), PERMANOVA (permutational multivariate analysis of variance), and MRPP (multi-response permutation procedure) analyses using a “vegan” package and were visualized by Principal coordinates analysis (PCoA) using the “ggplot2” package of R software (v4.1.0). Canonical correspondence analysis (CCA) and variation partitioning analysis (VPA) were performed using the R package “vegan.” The linear discriminant analysis (LDA) effect size (LEfSe) analysis (https://huttenhower.sph.harvard.edu/galaxy/) was performed to analyze and characterize the bacterial differences and microbial predicted pathway functions among different groups.

The metagenome functional predictions based on 16S rRNA gene sequencing of bacterial communities were analyzed by the Phylogenetic Investigation of Communities by the Reconstruction of Unobserved States 1 (PICRUSt 1) method (31). The OTU table and representative sequences subsampled at a minimum number of sequences (12981) were selected for the functional annotation to KEGG ortholog groups (KO) based on KEGG databases. Significant differential predicted pathway abundances were then analyzed and visualized by statistical analysis of taxonomic and functional profiles (STAMP) software with a two-sided Welch's *t*-test (32). Pearson correlation between phenotypic variables was analyzed and visualized by R software (v4.1.0) using the “corrplot” package. Mantel test was performed to examine the linkage between phenotypic variables and microbial communities or microbial predicted pathways (33).

The co-occurrence patterns were constructed to visualize the correlations between bacterial communities and microbial predicted pathways. Firstly, only those bacterial OTUs and microbial predicted KOs pathways with an average relative abundance >0.1% across all samples were retained according to Hartman et al. (34). We then normalized the filtered bacterial OTUs and microbial predicted KOs pathways separately by the “trimmed means of M” (TMM) method, and the normalized counts were expressed as relative abundance counts per million (CPM) using the R package “edgeR.” The indicator species of the filtered bacterial OTUs and microbial predicted KOs pathways were analyzed using the R package “indicspecies.” Differential OTUs and KOs abundances among all the groups were also analyzed by likelihood ratio tests (LRT) using the R package “edgeR.” The differential OTUs and KOs at a value of *p* < 0.05 were defined as OTUs and KOs responsive. Treatment-sensitive OTUs and KOs (hereafter: tsNodes) were then confirmed by both indicator species analysis and LRT. The TMM-normalized CPM counts of bacterial OTUs and microbial-predicted KOs pathways were then combined and further calculated the Spearman rank correlations by the R package “Hmisc.” Significant correlations (ρ > 0.7 and FDR-adjusted *p* < 0.01) were kept as the edges of the co-occurrence networks. Then, the co-occurrence networks were visualized using the *Fruchterman-Reingold* layout by the R package “igraph.” The network topological properties and modules were also calculated and identified to describe the complex patterns of the interrelationships. Keystone OTUs and KOs were identified as those nodes within top 1% of node degree values in the networks.

### Statistical Analysis

Data on growth performance and ileal histomorphology analysis were assessed using SPSS™ software (SPSS Inc., Chicago, IL, USA) by one-way ANOVA, and the contrast of means was evaluated by Tukey's multiple range tests. The data on SCFAs analysis were analyzed by two-tailed Student's *T*-test using SPSS™ software.

## Results

### Growth Performance

Performance results showed that, compared with the control group, *C. perfringens* infection significantly (*p* < 0.05) decreased the body weight and feed conversion and significantly (*p* < 0.05) increased bursal weight index of broilers, whereas dietary with *P. polymyxa* or *L. plantarum* significantly (*p* < 0.05) ameliorated *C. perfringens*-induced side effects of growth performance (body weight and feed conversion) (Table 1). Additionally, *L. plantarum* treatment also attenuated *C. perfringens*-induced enlarged bursa of fabricius (*p* < 0.05).

**Table 1:** Effect of probiotics on growth performance and immune organ indexes of broilers infected with *C. perfringens*.

**Items**	**Control**	**Cp**	**BSC10+Cp**	**Lac16+Cp**	**SEM**	***P*-value**
**Body weight (g)**						
Day 1	48.03	48.13	47.43	48.16	0.42	0.67
Day 21	637.88^a^	615.15^b^	648.48^a^	636.97^a^	8.33	0.016
**Day 1–21**						
BWG (g/d)	29.49^ab^	28.35^b^	30.05^a^	29.44^ab^	0.40	0.011
FI (g/d)	52.82	55.11	54.18	52.68	0.87	0.18
FCR	1.79^b^	1.94^a^	1.80^b^	1.79^b^	0.04	0.01
**Immune organ indexes**						
Spleen	0.85	1.02	0.9	1.11	0.096	0.22
Bursa of fabricius	1.87^b^	2.55^a^	2.3^a^	1.62^b^	0.25	<0.001

a, b *Mean values within a row with no common superscript differ significantly (p <0.05). SEM, standard error of mean; BWG, body weight gain; FI, feed intake; FCR, feed conversion ratio. n = 6 samples*.

### Morphological Observation

As presented in Table 2, *C. perfringens* infection significantly (*p* < 0.05) decreased the villus height and significantly (*p* < 0.05) increased the histopathological score of the ileum compared with those of the uninfected birds. Dietary with *P. polymyxa* or *L. plantarum* significantly (*p* < 0.05) ameliorated *C. perfringens*-induced ileal mucosa injury, as evidenced by the significantly (*p* < 0.05) increased villus height and villus height to crypt depth ratio, and decreased crypt depth and the histopathological score of the ileum.

**Table 2:** The ileal histomorphology of broilers infected with *C. perfringens*.

**Items**	**Control**	**Cp**	**BSC10+Cp**	**Lac16+Cp**	**SEM**	***P*-value**
Villus height (μm)	444.50^c^	382.85^d^	529.27^b^	627.77^a^	38.11	<0.001
Crypt depth (μm)	118.01^a^	121.31^a^	118.29^a^	96.24^b^	6.22	0.009
Villus/crypt ratio	3.79^bc^	3.19^c^	4.50^b^	6.59^a^	0.55	<0.001
Histopathological score	0.17^c^	3.00^a^	1.33^b^	1.17^b^	0.46	<0.001

a, b, c, d *Mean values within a row with no common superscript differ significantly (p <0.05). SEM, standard error of mean. n = six samples*.

**Figure 1:**
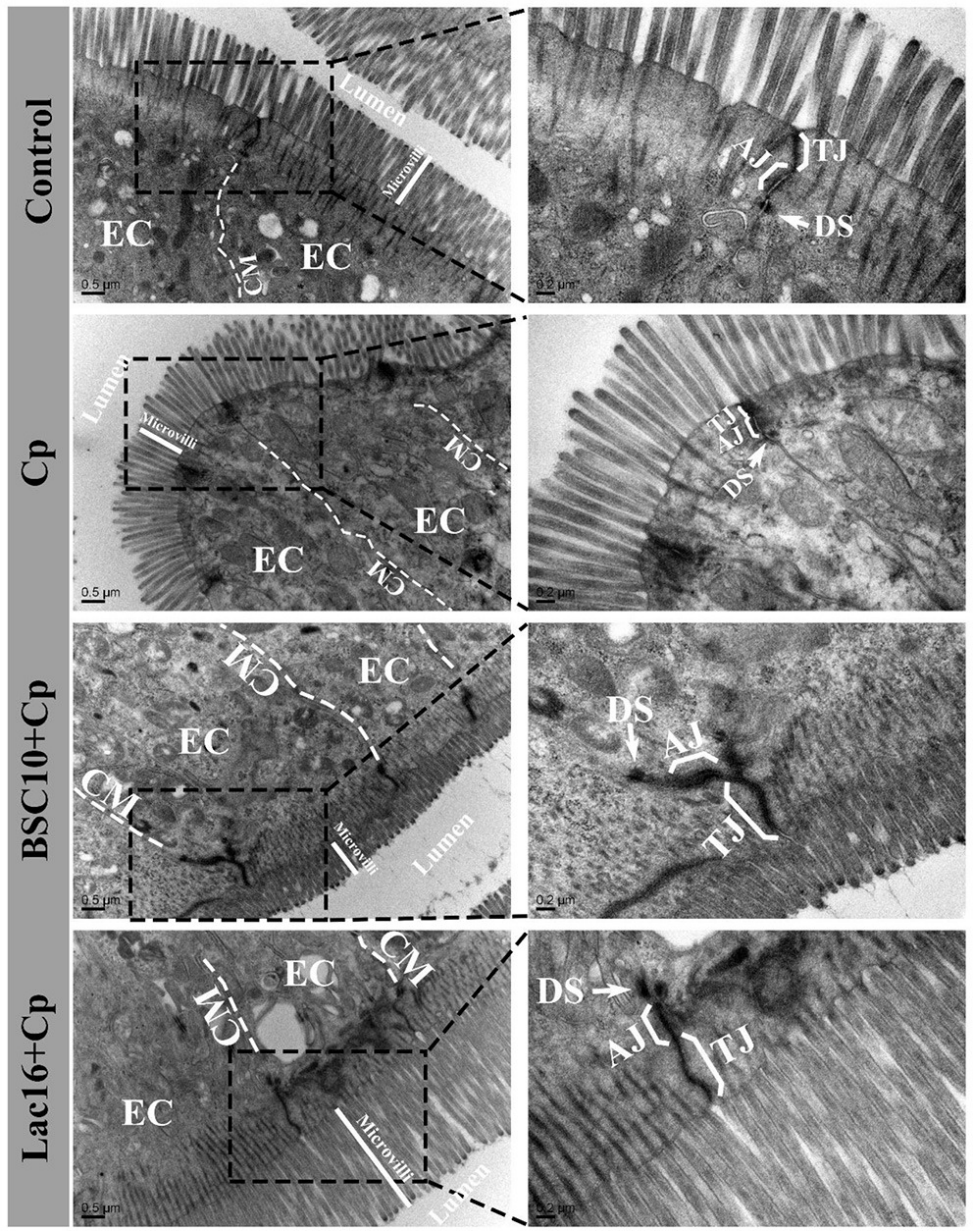
Transmission electron micrographs of the ileal microvilli in broilers at day 21 of age. EC, epithelial cell; CM, cell membrane; TJ, tight junction; AJ, adherens junction. The white arrow indicates desmosomes (DS).

TEM results showed that, compared with the control group, the ileum of the *C. perfringens*-infected birds showed sparse microvilli, disrupted and shorter tight junction, adherens junction, and desmosomes (Figure 1). Compared with the *C. perfringens*-infected group, the improved ileal intercellular junctional complexes of broilers in BSC10+Cp and Lac16+Cp groups were observed as evidenced by higher and ordered microvillus, longer tight junction and adherens junction, and darker desmosomes.

### SCFAs in Ileal Digesta

**Figure 2:**
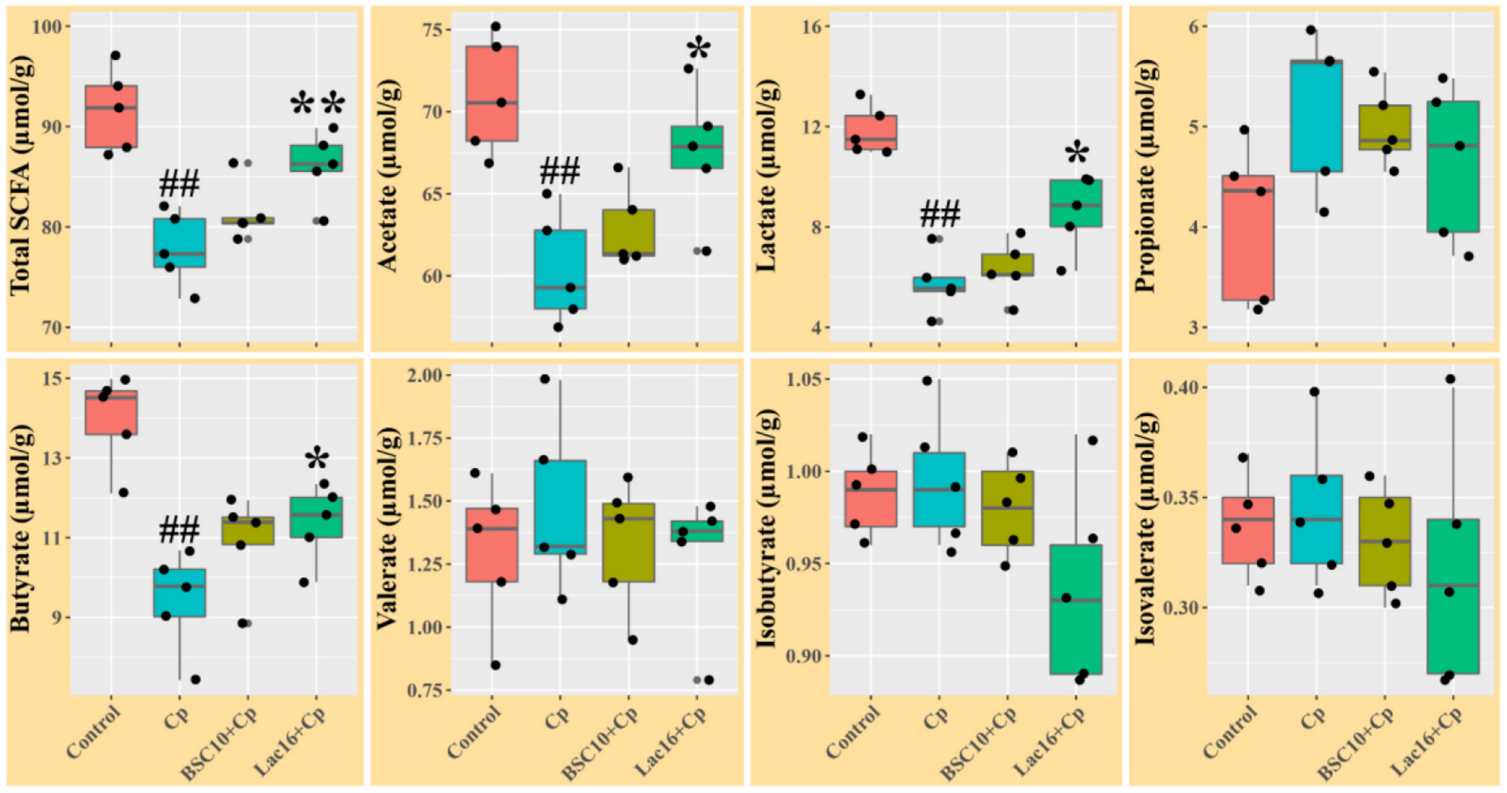
Short chain fatty acids levels in ileal digesta of broilers at day 21 of age. Significant differences vs. the control group: ^*##*^*p* < 0.01. Significant differences vs. the Cp group: *p < 0.05; **p < 0.01. *n* = five samples.

The levels of total SCFAs, acetate, lactate, and butyrate in ileal digesta of the *C. perfringens*-infected broilers were significantly (*p* < 0.01) decreased compared with the uninfected broilers (Figure 2). Compared with the *C. perfringens*-infected group, *P. polymyxa* treatment increased the concentrations of total SCFAs, acetate, lactate, and butyrate in ileal digesta of the *C. perfringens*-infected broilers but had no significant differences (*p* > 0.05). Dietary with *L. plantarum* significantly (*p* < 0.05) increased the contents of total SCFAs, acetate, lactate, and butyrate in ileal digesta of the *C. perfringens*-infected broilers.

### Microbial Composition

**Figure 3:**
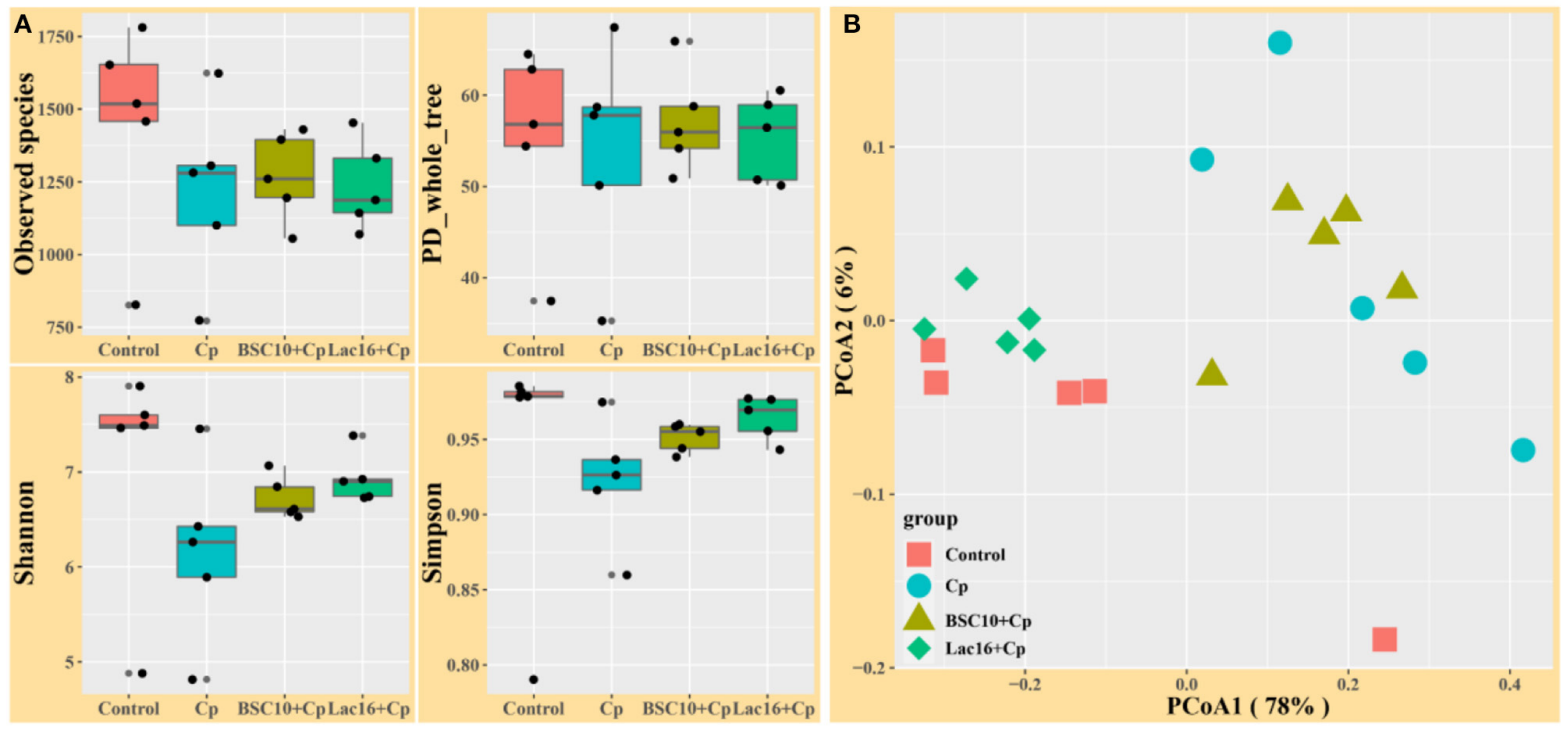
Diversity analyses of microbial communities among groups. **(A)** Alpha diversity analysis **(B)** Principal coordinates analysis (PCoA) based on Bray-Curtis distance. *n* = five samples.

Alpha diversity analysis showed that no significant difference was observed among the four groups (*p* > 0.05, Figure 3A). PCoA based on Bray-Curtis distance showed that the microbial communities were clustered into two different types of communities (Figure 3B). The microbial communities of broilers in control and Lac16+Cp groups formed a cluster and formed another cluster in the Cp and BSC10+Cp groups. Significant differences in beta diversity of microbial communities among all treatments were further confirmed by ANOSIM, PERMANOVA, and MRPP analyses (Table 3).

**Table 3:** ANOSIM, PERMANOVA, and MRPP analysis of microbial diversity among different treatments.

	**Anosim**		**Permanova**		**Mrpp**	
	***R***	***P***	***R^**2**^***	***P***	***A***	***P***
Treatment	0.561	0.001	0.597	0.002	0.327	0.001
Control vs. Cp	0.492	0.020	0.439	0.034	0.217	0.022
Cp vs. BSC10+Cp	0.084	0.232	0.109	0.376	0.018	0.275
Cp vs. Lac16+Cp	0.928	0.005	0.720	0.007	0.425	0.007

*ANOSIM, analysis of similarities; PERMANOVA, permutational multivariate analysis of variance; MRPP, multi-response permutation procedure. n = five samples*.

**Figure 4:**
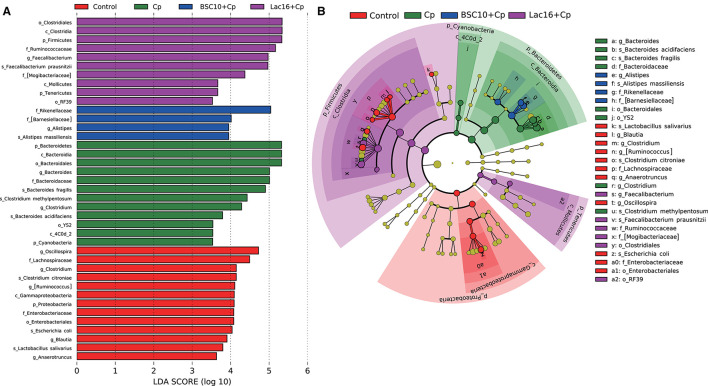
Linear discriminant analysis (LDA) effect size (LEfSe) analysis (LDA > 3., *p* < 0.05) of the intestinal microbes. Histogram **(A)** and cladogram **(B)** of LEfSe analysis among the four groups. The prefixes “p_,” “c_,” “o_,” “f_,” “g_,” and “s_” represent the annotated levels of phylum, class, order, family, genus, and species.

**Figure 5:**
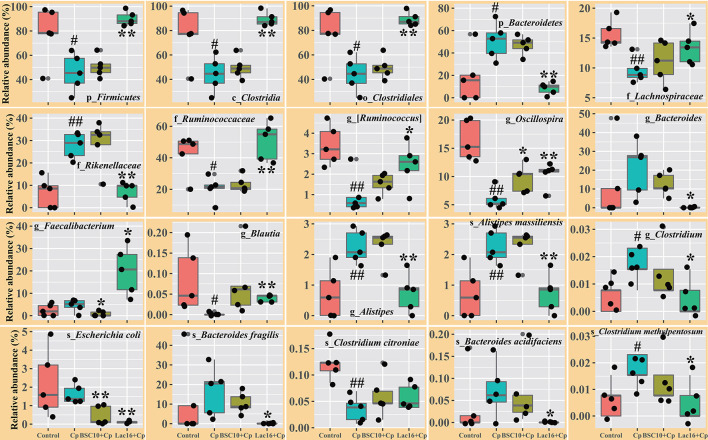
Comparison of the intestinal microbiota among the groups. Significant differences vs. the control group: ^#^*p* < 0.05; ^*##*^*p* < 0.01. Significant differences vs. the Cp group: *p < 0.05; **p < 0.01. The prefixes “p_,” “c_,” “o_,” “f_,” “g_,” and “s_” represent the annotated level of phylum, class, order, family, genus, and species. *n* = five samples.

LEfSe analysis was employed to explore the specific microbial features among the four groups (Figure 4). It was found that 36 taxa biomarkers in four groups were identified with LDA score >3, which mainly belong to the phyla of *Firmicutes, Bacteroidetes, Proteobacteria, Tenericutes*, and *Cyanobacteria*. Differential analysis results (Figure 5) further showed that, compared with the uninfected group, *C. perfringens* infection significantly (*p* < 0.05 or *p* < 0.01) reduced the relative abundances of *Firmicutes, Clostridia, Clostridiales, Lachnospiraceae, Ruminococcaceae*, [*Ruminococcus*], *Oscillospira, Blautia*, and *Clostridium citroniae* in the cecum, while markedly (*p* < 0.05 or *p* < 0.01) increased the relative abundances of *Bacteroidetes, Rikenellaceae, Alistipes, Alistipesmassiliensis, Clostridium*, and *Clostridium methylpentosum*. Compared with the *C. perfringens*-infected birds, the significantly (*p* < 0.05 or *p* < 0.01) increased *Oscillospira* abundance and decreased relative abundances of *Faecalibacterium*, and *Escherichia coli* were observed in the broilers of the BSC10+Cp group. Furthermore, *L. plantarum* significantly (*p* < 0.05 or *p* < 0.01) improved the anomalous microbial composition induced by *C. perfringens* infection, as evidenced by the increased relative abundances of *Firmicutes, Clostridia, Clostridiales, Lachnospiraceae, Ruminococcaceae*, [*Ruminococcus*], *Oscillospira, Faecalibacterium*, and *Blautia* and the reduced relative abundances of *Bacteroidetes, Rikenellaceae, Bacteroides, Alistipes, Alistipesmassiliensis, Clostridium, Escherichia coli, Bacteroides fragilis, Bacteroides acidifaciens, Clostridium methylpentosum* (Figure 5).

### Metagenome Functional Predictions

**Figure 6:**
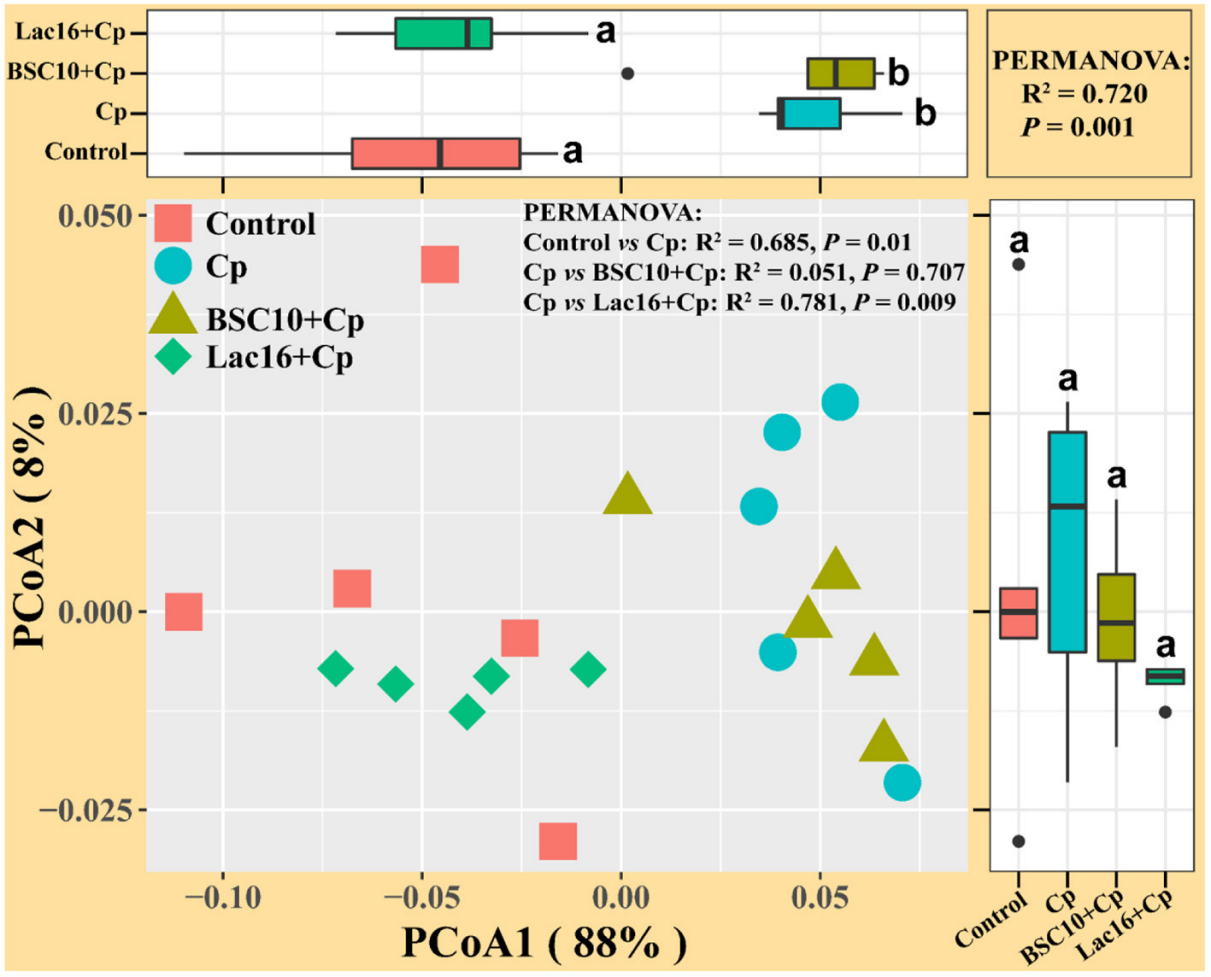
Principal coordinates analysis (**PCoA**) of predicated metabolic functions among groups based on Bray-Curtis distance. ^a,b^indicate a significant difference (*p* < 0.05).

**Figure 7:**
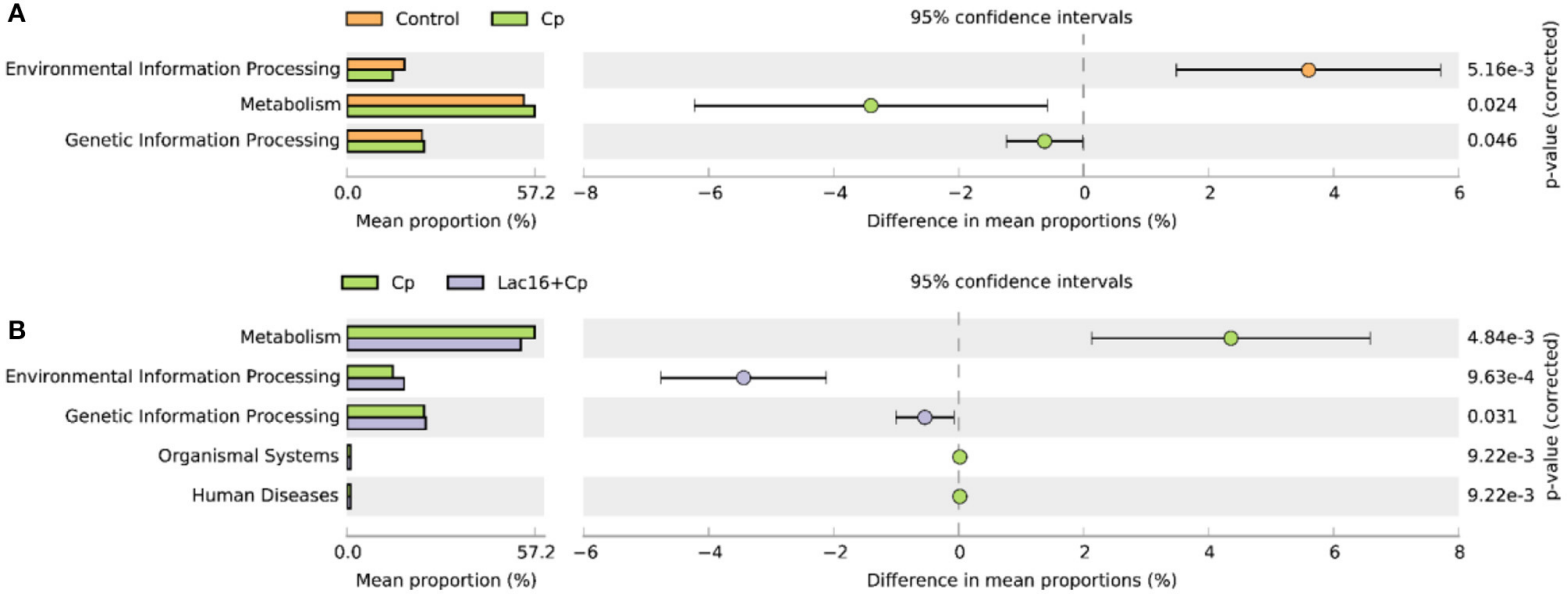
Comparison of predicted metabolic pathway abundances between the groups by statistical analysis of taxonomic and functional profiles (**STAMP**) at Level 1. **(A)** Control vs. Cp; **(B)** Cp vs. Lac16+Cp.

**Figure 8:**
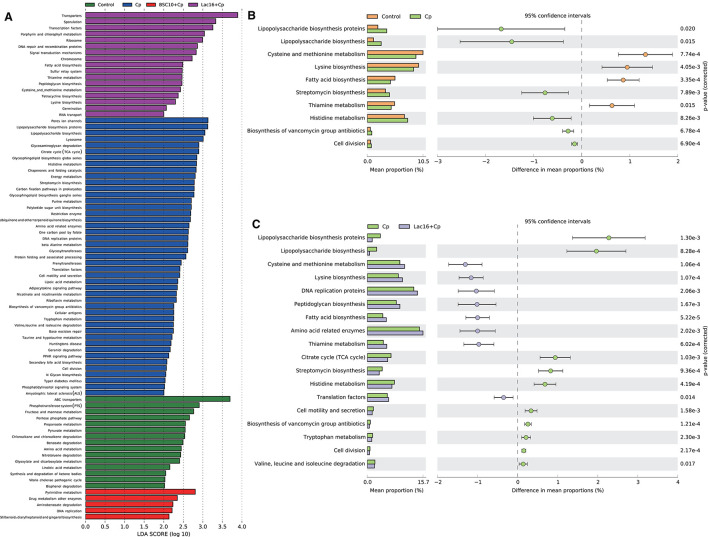
**(A)** LEfSe analysis (LDA > 2., *p* < 0.01) of the microbial-predicted metabolic pathway functions at Level 3. **(B,C)** Comparison of predicted pathway abundances between the groups by statistical analysis of taxonomic and functional profiles (**STAMP**) at Level 3. **(B)** Control vs. Cp; **(C)** Cp vs. Lac16+Cp.

PICRUSt analysis was performed to explore the metagenome functions based on 16S rRNA marker gene sequences. PCoA results based on Bray-Curtis distance showed that the microbial metabolic functions were significantly distinct among the groups (Figure 6). Consisted with PCoA results for microbial communities, *C. perfringens* infection also altered the bacterial metabolic functions, whereas dietary with *L. plantarum* significantly (*R*^2^ = 0.781, *P* = 0.009) ameliorated the shifts of bacterial metabolic functions induced by *C. perfringens* infection. The STAMP and LEfSe analysis based on level 1 and level 3 of the microbial-predicted pathway functions further verified the differences in metabolic functions (Figures 7, 8 and Supplementary Figure 2). Specifically, *L. plantarum* treatment significantly (*p* < 0.05) inversed the shifts of bacterial metabolic functions induced by *C. perfringens* infection, such as metabolism, lipopolysaccharide (LPS) biosynthesis, LPS biosynthesis proteins, fatty acid biosynthesis, streptomycin biosynthesis, biosynthesis of vancomycin group antibiotics, cysteine and methionine metabolism, lysine biosynthesis, thiamine metabolism, histidine metabolism, and cell division (Figure 8 and Supplementary Figure 2). Dietary with *P. polymyxa* had less effects on microbial metabolic functions than *L. plantarum* in *C. perfringens*-infected broilers (Figure 8 and Supplementary Figure 2).

### Relationships Between Phenotypic Variables, Bacterial Communities and Microbial Metabolic Functions

**Figure 9:**
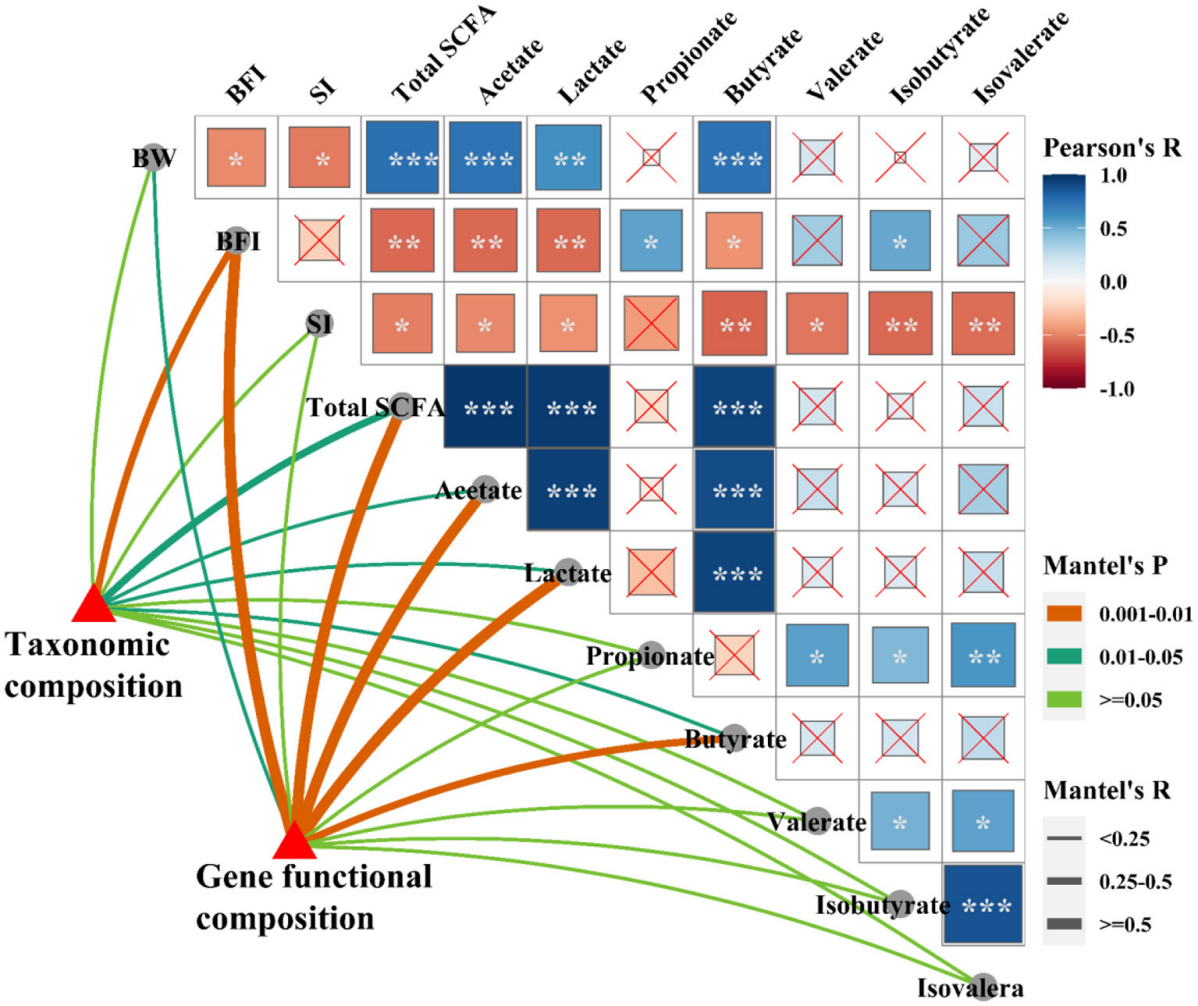
Relationships among phenotypic variables, the microbial community, and metabolic functional composition. Pairwise comparisons of phenotypic variables with a color gradient denoting Pearson correlation coefficient. Taxonomic and functional community structures were related to each phenotypic variable by Mantel correlation (based on Bray-Curtis dissimilarity). The edge width represents the Mantel's r statistic for the corresponding distance correlations, and edge color denotes the statistical significance. *p < 0.05; **p < 0.01; ***p < 0.001.

The shifts in microbial community and metabolic functional composition induced by *C. perfringens* infection and dietary with probiotics were tightly linked to phenotypic variables and ileal SCFAs as revealed by the Mantel test (Figure 9), CCA and VPA analyses (Supplementary Figure 3). Pearson correlation analysis (Figure 9) showed that the ileal SCFAs (including total SCFAs, acetate, lactate and butyrate) were positively (*p* < 0.001) correlated with the final body weight, whereas were negatively (*p* < 0.05 or *p* < 0.01) correlated with immune organ indexes (spleen and bursa of Fabricius). The bursa of Fabricius index was positively (*p* < 0.05) correlated with propionate and isobutyrate. Additionally, the spleen index was negatively (*p* < 0.05) correlated with valerate, isobutyrate, and isovalerate. Mantel correlation analysis (Figure 9) showed that taxonomic composition was significantly correlated with bursa of Fabricius index (0.25 < *r* < 0.5, 0.001 < *p* < 0.01), total SCFAs (0.25 < *r* < 0.5, 0.01 < *p* < 0.05), acetate (*r* < 0.25, 0.01 < *p* < 0.05), lactate (*r* < 0.25, 0.01 < *p* < 0.05), and butyrate (*r* < 0.25, 0.01 < *p* < 0.05). The microbial functional compositions were significantly correlated with the final body weight (*r* < 0.25, 0.01 < *p* < 0.05), bursa of Fabricius index (*r* > 0.5, 0.001 < *p* < 0.01), total SCFAs (*r* > 0.5, 0.001 < *p* < 0.01), acetate (*r* > 0.5, 0.001 < *p* < 0.01), lactate (*r* > 0.5, 0.001 < *p* < 0.01), and butyrate (0.25 < *r* < 0.5, 0.001 < *p* < 0.01). CCA analysis showed that bursa of Fabricius index (*R*^2^ = 0.56, *P* = 0.004; *R*^2^ = 0.55, *P* = 0.004), ileal total SCFAs (*R*^2^ = 0.58, *P* = 0.002; *R*^2^ = 0.68, *P* = 0.001), acetate (*R*^2^ = 0.53, *P* = 0.002; *R*^2^ = 0.67, *P* = 0.001), lactate (*R*^2^ = 0.66, *P* = 0.002; *R*^2^ = 0.65, *P* = 0.001), and butyrate (*R*^2^ = 0.51, *P* = 0.005; *R*^2^ = 0.72, *P* = 0.001) were significantly correlated with the bacterial (Supplementary Figure 3A) and microbial functional (Supplementary Figure 3B) community structures. The final body weight (*R*^2^ = 0.36, *P* = 0.026) and isobutyrate (*R*^2^ = 0.48, *P* = 0.002) were also significantly correlated with the microbial functional community structure (Supplementary Figure 3B). The VPA analysis showed that the phenotypic variables and ileal SCFAs explained 27.21 and 28.73% of the variations in the bacterial communities, and their interaction explained 10.72% of the variation (Supplementary Figure 3C). For the microbial predicted pathway functions, phenotypic variables and ileal SCFAs explained 17.07 and 25.26% of the variations, respectively, and the interaction explained 24.00% of the variation (Supplementary Figure 3D).

**Figure 10:**
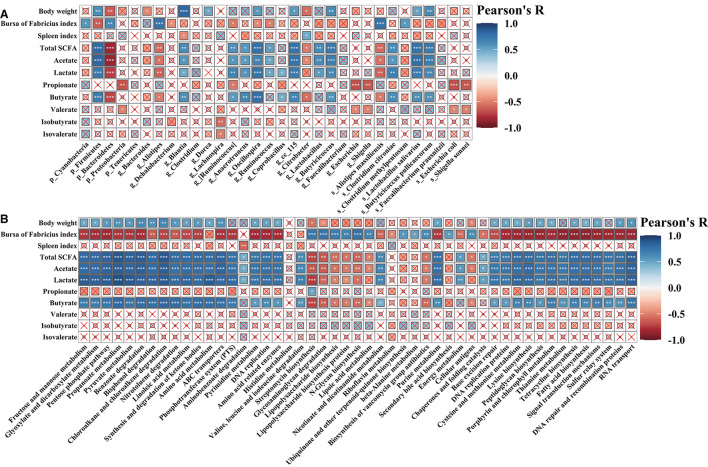
Pearson's correlation analysis. Correlation of phenotypic variables and microbial communities **(A)** and microbial-predicted metabolic pathway functions **(B)**. The color and the dot size represent the correlation coefficient. *p < 0.05; **p < 0.01; ***p < 0.001.

To further investigate the specific relationships between phenotypic variables and the microbial community or microbial metabolic functional composition, Pearson's correlation analysis was performed (Figure 10). As shown in Figure 10A, we found that the final body weight was positively (*p* < 0.05, *p* < 0.01, or *p* < 0.001) correlated with cecal *Firmicutes, Blautia, Dorea, Oscillospira*, cc_115, *Lactobacillus, Lactobacillus salivarius*, and *Butyricicoccus pullicaecorum* but negatively correlated with *Bacteroides* and *Citrobacter*. The bursa of Fabricius index was positively (*p* < 0.05, *p* < 0.01, or *p* < 0.001) correlated with cecal *Cyanobacteria, Bacteroidetes, Alistipes, Alistipes massiliensis*, and *Clostridium methylpentosum*, whereas negatively correlated with *Firmicutes, Dehalobacterium*, [*Ruminococcus*], *Oscillospira*, and *Ruminococcus*. The ileal SCFAs (such as total SCFAs, acetate, lactate, and butyrate) were positively (*p* < 0.05, *p* < 0.01, or *p* < 0.001) correlated with cecal *Firmicutes, Blautia*, [*Ruminococcus*], *Anaerotruncus, Oscillospira, Ruminococcus, Coprobacillus*, cc_115, *Lactobacillus, Butyricicoccus, Clostridium citroniae, Lactobacillus salivarius*, and *Butyricicoccus pullicaecorum*, whereas negatively (*p* < 0.05, *p* < 0.01, or *p* < 0.001) correlated with *Bacteroidetes, Alistipes, Citrobacter*, and *Alistipes massiliensis*. The ileal propionate and valerate were positively (*p* < 0.05, *p* < 0.01, or *p* < 0.001) correlated with cecal *Proteobacteria, Escherichia, Shigella, Escherichia coli*, and *Shigella sonnei*. As shown in Figure 10B, the results showed that the final body weight and ileal SCFAs (including total SCFAs, acetate, lactate, and butyrate) were positively (*p* < 0.05, *p* < 0.01, or *p* < 0.001) correlated with cecal microbial carbohydrate metabolism, amino acid metabolism, peptidoglycan biosynthesis, fatty acid biosynthesis, and tetracycline biosynthesis, whereas negatively (*p* < 0.05, *p* < 0.01, or *p* < 0.001) correlated with glycan biosynthesis and metabolism (such as LPS biosynthesis and LPS biosynthesis proteins) and antibiotic biosynthesis (streptomycin biosynthesis and biosynthesis of vancomycin group antibiotics). The bursa of Fabricius index exerts opposite effect on the correlation with the cecal microbial metabolic functions compared with that of final body weight.

### Microbial Co-occurrence Patterns

**Figure 11:**
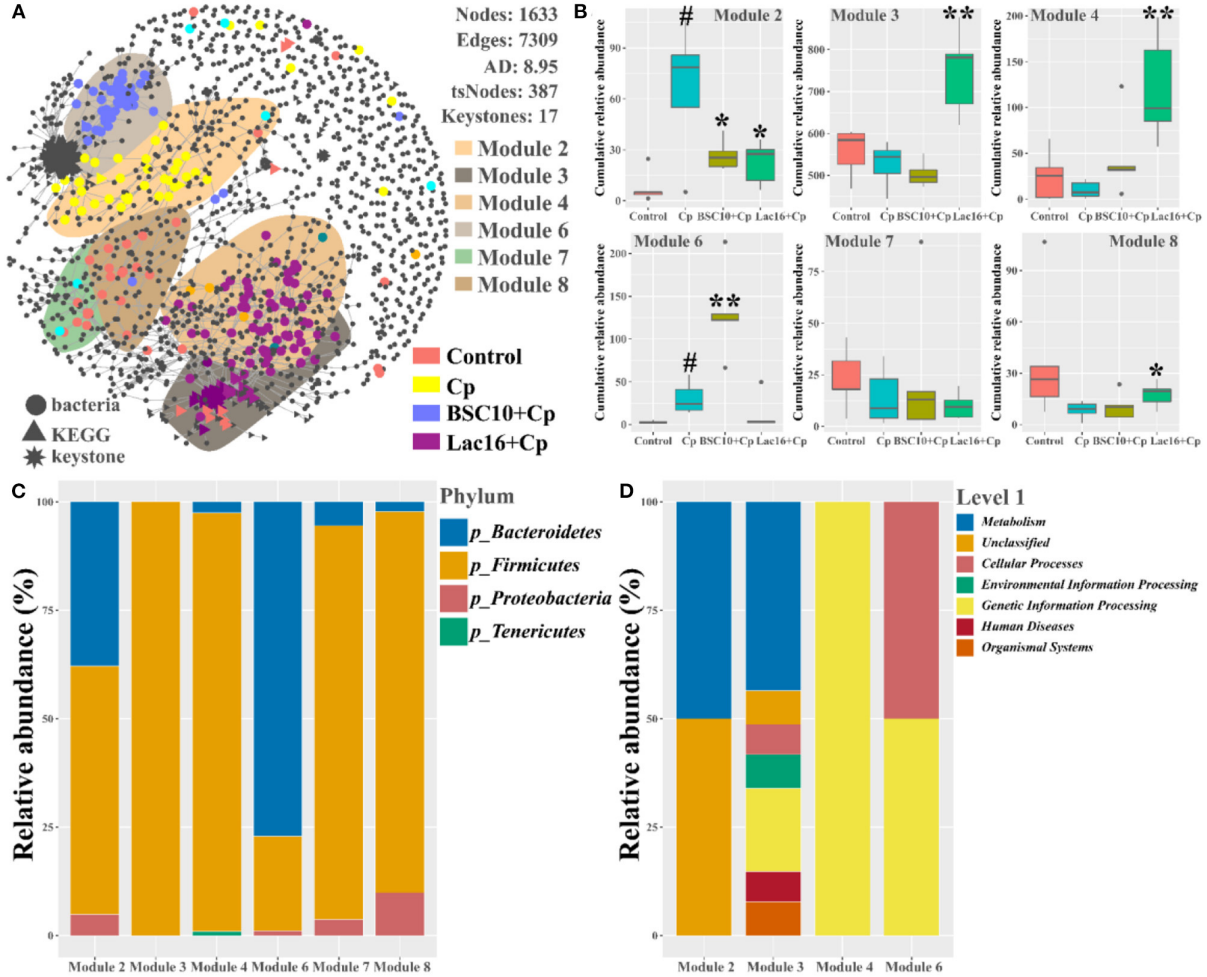
Co-occurrence network of bacterial and metabolic functional communities. **(A)** Significant and close (|ρ| >0.8, *p* < 0.05) correlations among bacterial OTUs and metabolic KOs are visualized in the network with gray lines. The bacterial, metabolic functional, and keystone nodes are indicated by circles, triangles, and stars, respectively. Sensitive nodes are colored by their association with the treatments, and gray nodes are insensitive to the treatments. Shaded areas represent the top six modules with high proportions. **(B)** Cumulative-relative abundance (as counts per million, CPM; y-axis in × 1,000) of the bacterial OTUs metabolic KOs belonging to the responsive modules. **(C)** The bacterial phyla and metabolic functional compositions for each module. Significant differences versus Control group: ^#^*p* < 0.05. Significant differences versus Cp group: *p < 0.05; **p < 0.01.

Next, we explored the distribution patterns of sensitive OTUs and KOs in the co-occurrence patterns of bacterial communities and microbial metabolic functions in four groups (Figure 11 and Table 4). The co-occurrence network consisted of 1,633 nodes and 7,309 edges (with a mean of 8.95 edges per node) (Figure 11A). In the network, the bacteria shared 1,368 nodes with 2,204 edges (with a mean of 3.22 edges per node), and the microbial metabolic functions shared 265 nodes with 4,027 edges (with a mean of 30.39 edges per node). Among the 1,633 nodes, 387 nodes (235 tsOTUs and 150 tsKOs) were identified as treatment-sensitive nodes, and 17 nodes (three OTUs and 14 KOs) were defined as keystones (Table 4 and Supplementary Tables 2, 3). Consistent with the results showed in Figures 5, 8, compared with the uninfected group, *C. perfringens* infection significantly (*p* < 0.05 or *p* < 0.01) increased the relative abundance of 15 keystones, such as *Rikenellaceae* and microbial metabolisms (LPS biosynthesis, LPS biosynthesis proteins, streptomycin biosynthesis, etc.) (Figure 4). Compared with the *C. perfringens*-infected group, dietary with *L. plantarum* significantly (*p* < 0.05 or *p* < 0.01) decreased *C. perfringens*-induced relative abundance of 17 keystones.

**Table 4:** Topological properties of the co-occurrence network.

**Community**	**Bacteria**	**KEGG**
Nodes	1,368	265
Edges	2,204	4,027
Interactive edges	1,078	
Average degree	3.22	30.39
sNodes	235	152
Keystones	3	14

In addition, we noticed that the distribution patterns of microbial and microbial functional associations also responded to treatments. Six modules with relatively high proportions in the network were identified and visualized (Figures 11A,B). We found that the type of sensitivity of these modules to the specific-treated groups (Figures 11A,B), and their distribution in the network partially reflected the community similarity showed in Figures 3B, 6. For example, the effect of *C. perfringens* infection in the cecal microbial and functional communities was apparent with discrete modules (Modules 2 and 6) in the network (Figure 11B). Module 2 and Module 6 were separated from other modules that primarily contained sensitive OTUs and KOs specific to the control (Modules 7 and 8) and Lac16+Cp (Module 3 and Module 4) groups. The cumulative-relative abundances of the sensitive nodes of Module 2 in the BSC10+Cp and Lac16+Cp groups were significantly (*p* < 0.05) lower than those in the *C. perfringens*-infected group. The cumulative-relative abundances of the sensitive nodes of three modules (Modules 3, 4, and 8) in the Lac16+Cp group and Module 6 in the BSC10+Cp group were significantly (*p* < 0.05 or *p* < 0.01) higher than those in the *C. perfringens*-infected group. Furthermore, we found that the six responsive modules comprised a broad set of bacteria and microbial metabolic functions (Figure 11C), indicating that the different treatments may be not target-specific microbial lineages.

## Discussion

Necrotic enteritis caused by *C. perfringens* in poultry exists in two forms: clinical (acute) or subclinical (chronic) infections. Subclinical necrotic enteritis infection accompanies with continuously chronic damage of the gastrointestinal mucosa, which leads to poor growth performance (decreased digestion and absorption, reduced body weight and the feed-conversion ratio) of broilers with or without mortality (9, 35). Subclinical necrotic enteritis without mortality in the present study was successfully induced by *C. perfringens* infection as observed by decreased body weight and feed conversion, and increased bursal weight index of broilers. The result was consistent with previous studies that subclinical necrotic enteritis infection impaired broiler growth performance with no mortality (36, 37). Our previous study reported that *Saccharomyces boulardii* attenuates *C. perfringens*-induced inflammatory response *via* the TLR4/TLR15-MyD88-signaling pathway in HD11 avian macrophages (23). *Bacillus amyloliquefaciens* could alleviate necrotic enteritis-induced undesirable effects by modulation of genes related to gut integrity, apoptosis, and immunity, hence further improve performance (38). Although many studies have reported that *Lactobacillus* or *Bacillus* could attenuate *C. perfringens*-induced necrotic enteritis (24–26), but the potential protective mechanism of probiotics against *C. perfringens* infection remains poorly understood, which needs to be further investigated. This study shows that dietary with *L. plantarum* or *P. polymyxa* significantly attenuates *C. perfringens* infection-induced compromise of growth performance. Previous studies showed that *C. perfringens* infection compromised the growth performance of broilers by impairing intestinal health and inducing intestinal dysbacteriosis (24, 39). The ameliorated growth performance of *C. perfringens*-infected broilers in *L. plantarum* or *P. polymyxa*-fed groups in this study may be related to the improved intestinal health and intestinal microbiota.

Healthy intestinal morphology and balance of intestinal microbes are important indicators of gastrointestinal tract homeostasis (40, 41). Due to the continuously chronic intestinal mucosa damage with low or no mortality, subclinical necrotic enteritis imposes a huge economic burden on global poultry industry (9). In this study, the increased ileal crypt depth and the histopathological score and the decreased ileal villus height and villus height to the crypt-depth ratio were observed in *C. perfringens*-infected broilers, which was the main reason to impair the growth performance mentioned above and also indicated that subclinical necrotic enteritis was successfully induced (35). It was found that *C. perfringens*-induced ileal mucosa damage was alleviated by dietary with *P. polymyxa* or *L. plantarum*, as illustrated by increased ileal villus height and villus height to the crypt-depth ratio and decreased ileal crypt depth and the histopathological score. Previous studies also reported that dietary with probiotics had positive effects on reducing subclinical necrotic enteritis occurrence by ameliorating *C. perfringens*-induced damage of intestinal mucosa (24, 42). The intact intestinal epithelium serves as a physical intestinal mucosal barrier function against invasion of zoonotic enteric pathogens and is responsible for proper nutrient absorption and utilization and waste secretion (43). This physical mucosal barrier is composed of junctional complexes, which provide different types of intercellular connections (44). It is reported that the junctional complexes are binding sites of *C. perfringens* toxins and enterotoxins (45, 46). In the present study, *C. perfringens*-infected broilers showed shorter and sparse ileal microvilli, disrupted and shorter tight junction, adherens junction, and desmosomes, consistent with previous studies (47, 48). The intestinal epithelial junction was enhanced in the increased ileal villus of broilers in BSC10+Cp and Lac16+Cp groups, as observed by higher and ordered microvillus, longer tight junction, enhanced adherens junction, and darker desmosomes. The improved intestinal morphology and mucosal barrier function in probiotics treatments may contribute to greater nutrient absorption and utilization from the intestinal digesta (25) and thereby attenuates impairment of growth performance in *C. perfringens*-infected broilers mentioned above.

SCFAs (mainly including acetate, propionate, and butyrate) are fermentative products metabolized by the gastrointestinal commensal microbiota from dietary carbohydrates (49, 50). As one of the major microbial metabolites, SCFAs play critical roles in maintaining or improving the integrity of intestinal epithelium and tissue repair after mucosal damage (50, 51). *C. perfringens* infection in this study decreased the levels of ileal total SCFAs, acetate, lactate, and butyrate of broilers, which might impair the energy supply for intestinal enterocytes to repair mucosal damage. Partially consistent with our findings, a previous study reported that subclinical necrotic enteritis infection decreased concentrations of cecal acetate and butyrate of broilers, but increased levels of cecal lactate and propionate partly because of the increased relative abundance of *Lactobacillus* (52). SCFAs exert not only as an energy source for the host but also as main regulators of the physiological function of intestinal epithelial cells and immune cells (50, 53). SCFAs, especially butyrate and lactate, are also important bacterial metabolites that exert antibacterial and anti-inflammatory activities (53–55). The altered concentrations of the ileal SCFAs (total SCFAs, acetate, lactate, and butyrate) induced by *C. perfringens* infection were significantly reversed in the Lac16+Cp group, but not significantly reversed in the BSC10+Cp group, indicating that the improved intestinal morphology and mucosal barrier function of *C. perfringens*-infected broilers in the *L. plantarum*-treated group may be partly due to the increased intestinal SCFAs levels.

The enteric pathogen infection-induced intestinal dysbiosis could aggravate the gastrointestinal mucosal injury and then compromise the intestinal health and growth performance (56). Many studies reported that dysbiosis is allegedly correlated with *C. perfringens* infection (14, 57, 58), but it remains unclear about the causal relationship between the intestinal dysbiosis and gastrointestinal infectious diseases (59). In this study, *L. plantarum* supplementation significantly improved *C. perfringens* infection-induced intestinal dysbiosis and bacterial metabolic dysfunctions. PCoA results clearly indicated that dietary *L. plantarum* significantly reversed *C. perfringens* infection induced shifts of structures of microbial communities (Figure 3B and Table 3) and bacterial metabolic functions (Figure 6), which is consistent with previous studies (60, 61). *C. perfringens* infection significantly induced a shift of broiler intestinal microbiota structure (61), which could be reversed by dietary *Bacillus* direct-fed microbial (DFM) in feed (60). The structural shifts may be related with the changes of microbial and microbial metabolic compositions. Dietary *L. plantarum* improved *C. perfringens* infection-induced anomalous microbial composition as evidenced by enriched *Firmicutes, Ruminococcaceae*, SCFAs-producing bacteria (*Lachnospiraceae, Ruminococcaceae, Oscillospira, Faecalibacterium*, and *Blautia*) (62–65), which may be partly related to the increase of bacterial fatty acid biosynthesis and intestinal SCFAs levels observed in this study. *L. plantarum* supplementation significantly reduced *Bacteroidetes*, drug-resistant bacteria (*Bacteroides, Alistipes*) (66, 67), and enteric pathogens (*Escherichia coli, Bacteroides fragilis*) (66), which may partly contribute to the inhibition of bacterial LPS biosynthesis and antibiotic biosynthesis (streptomycin and vancomycin). These findings indicated that *L. plantarum*-mediated-ameliorated compromise of growth performance in *C. perfringens*-infected broilers may be related to the restored gut microbial communities and bacterial metabolic functions, which should be further confirmed by whole shotgun metagenomic sequencing because of the limited taxonomical and functional attributes offered by 16S rRNA gene sequencing.

Strong correlations among the final body weight, ileal SCFAs (including total SCFAs, acetate, lactate, and butyrate), microbial communities, and microbial metabolic functions were observed. Consistent with previous reports (68, 69), the final body weight had a strong positive correlation with the ileal SCFAs (including total SCFAs, acetate, lactate, and butyrate), further indicating that the increased intestinal SCFAs levels were beneficial for ameliorating *C. perfringens* infection-induced compromise of broiler growth performance. Furthermore, the final body weight and ileal SCFAs (including total SCFAs, acetate, lactate, and butyrate) were positively correlated with SCFAs-producing bacteria (*Blautia, Oscillospira, Lactobacillus, Lactobacillus salivarius*, and *Butyricicoccus pullicaecorum*) and bacterial fatty acid biosynthesis, whereas negatively correlated with drug-resistant bacteria (*Bacteroides* and *Citrobacter*), which was partly consistent with previous findings (70–72). These results indicated that the body weight and intestinal SCFAs might be principal factors affecting the bacterial communities and microbial metabolic functions.

Co-occurrence patterns of gut microbiota were further performed to investigate the treatment-sensitive species, keystones, and the microbial interactions. The results found that 235 bacteria and 150 microbial metabolic functions were sensitive to the four treatments, and most of the tsNodes significantly grouped in six distinct modules that reflected the different treatments. The uninfected treatment-sensitive nodes grouped in Modules 7 and 8; *C. perfringens*-infected-treatment-sensitive nodes grouped in Modules 2 and 6; *P. polymyxa* pretreatment-sensitive nodes grouped in Module 6; *L. plantarum* pretreatment-sensitive nodes grouped in Modules 3 and 4, indicating that dietary *L. plantarum* significantly modulated the co-occurrence interactions of intestinal microbes. By frequently interacting with many other microbes, keystone species are thought to play crucial roles in modulating microbial communities and functions (34, 73). The present study found that three bacteria (*Rikenellaceae, Bacteroides, Bacteroides*_unidentified) and 14 bacterial metabolic functions (LPS biosynthesis, LPS biosynthesis proteins, streptomycin biosynthesis etc.) were defined as keystones, in which dietary *L. plantarum* significantly decreased *C. perfringens* infection induced all the 17 keystones. *Bacteroides* species are multiple drug-resistant and significant clinical anaerobic pathogenic bacteria that can cause life-threatening infection with mortality of more than 19% (66). As the major component of the outer membrane of gram-negative microbes, LPS acts as a key pathogenic stimulator for the dysfunctions and plays major role in pathogens-mediated toxicity and pathogenicity (74). These results indicate that the shifts of these keystones maybe the driving factors involved in *L. plantarum*-mediated-ameliorated growth performance and improved mucosal damage of the *C. perfringens*-infected broilers.

## Conclusion

The current results indicate that dietary *L. plantarum*-mediated amelioration of growth performance and improvement of intestinal mucosal damage of broilers under subclinical NE condition are associated with the increased intestinal SCFAs levels, enhanced intestinal epithelial barriers, and improved intestinal dysbiosis. Dietary *P. polymyxa*-mediated amelioration of broiler growth performance under subclinical NE condition may partly be due to the improved mucosal structure and intestinal epithelial barriers. These findings provide a potential preventive approach against avian necrotic enteritis caused by *C. perfringens* infection. However, the detailed preventive mechanism of *L. plantarum* against subclinical necrotic enteritis should be further investigated.

## Data Availability Statement

Raw sequences have been deposited in the Genome Sequence Archive (GSA) of the BIG Data Center (https://bigd.big.ac.cn/gsa/) under accession number PRJCA004271/CRA003760.

## Ethics Statement

All the procedures of this project were conducted according to the Chinese guidelines for animal welfare and were approved by the Zhejiang University Institutional Animal Care and Use Committee (Permission number: ZJU20160416).

## Author Contributions

WL and HZ conceptualization and supervision. BW, YM, and YZ data curation, microbial analysis, writing—original draft, review, and editing. BW, QG, and YZ conducted the animal experiments. LG, XL, SX, and FW assisted with the experiments. XL and SX assisted in the manuscript preparation. All authors contributed to the article and approved the submitted version.

## Funding

This study was supported by the National Natural Science Foundation of China (Grant Nos. 31472128 and 31672460), the Natural Science Foundation of Zhejiang province (Grants Nos. LZ20C170002 and 2006C12086), and National High-Tech R&D Program (863) of China (Grant No. 2013AA102803D).

## Conflict of Interest

The authors declare that the research was conducted in the absence of any commercial or financial relationships that could be construed as a potential conflict of interest.

## Publisher's Note

All claims expressed in this article are solely those of the authors and do not necessarily represent those of their affiliated organizations, or those of the publisher, the editors and the reviewers. Any product that may be evaluated in this article, or claim that may be made by its manufacturer, is not guaranteed or endorsed by the publisher.

## Supplementary Material

Figure S1 Probiotics exerts anti-*C. perfringens* activity. **(A)** Fermented supernatant of *P. polymyxa* (BSC10) and *L. plantarum* (Lac16) significantly inhibited the expression of virulence genes (α and β toxins) of *C. perfringens*. Significant differences versus control group: ^*^*p* < 0.05. **(B)** BSC10 and Lac16 cultures significantly inhibited *C. perfringens* growth. **(C)** Live Lac16 significantly protect *C. elegans* against *C. perfringens* infection.

Figure S2 Comparison of predicted pathway abundances between the groups by statistical analysis of taxonomic and functional profiles (STAMP). **(A)** Control versus Cp; **(A)** Cp versus BSC10+Cp; **(B)** Cp versus Lac16+Cp.

Figure S3 Canonical correspondence analysis and variation partitioning analysis of the phenotypic variables (including body weight, bursa of fabricius index and spleen index) and ileal SCFAs for the bacterial community **(A,C)** and microbial predicted pathway functions **(B,D)**.

Figure S4 Relative abundances of the keystone species. Significant differences versus Control group: ^#^*p* < 0.05; ^*##*^*p* < 0.01. Significant differences versus Cp group: ^*^*p* < 0.05; ^**^*p* < 0.01. The prefixes “b_” and “k_” represent the bacteria and KEGG. *n* = 5 samples.
