# Supplementary figures and images for: Dietary Supplementation With Lactobacillus plantarum Ameliorates Compromise of Growth Performance by Modulating Short-Chain Fatty Acids and Intestinal Dysbiosis in Broilers Under Clostridium perfringens Challenge
Source: Front Nutr. 2021 Oct 14;8:706148. doi: 10.3389/fnut.2021.706148 (PMC8551491; doi:10.3389/fnut.2021.706148)

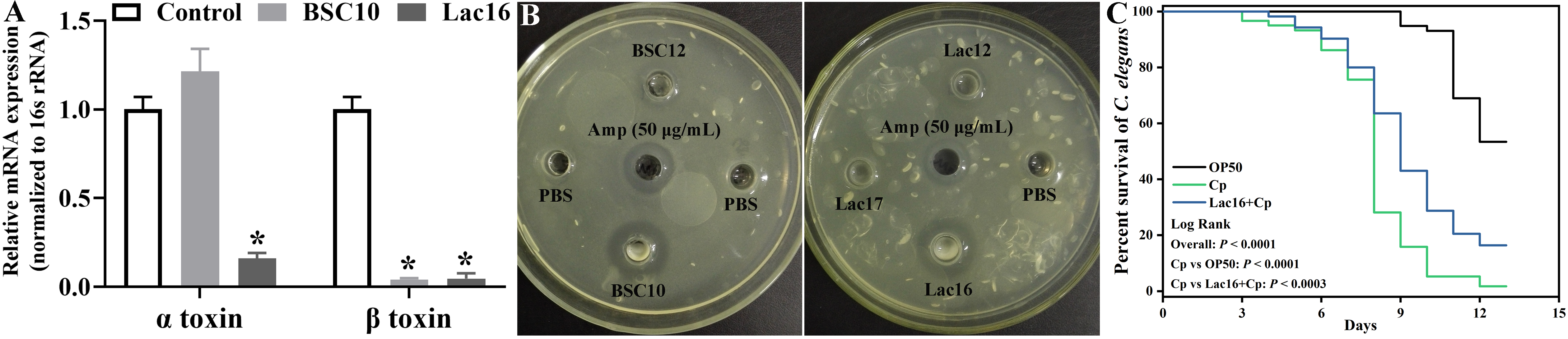

Supplement: Figure S1 — Probiotics exerts anti-C. perfringens activity. (A) Fermented supernatant of P. polymyxa (BSC10) and L. plantarum (Lac16) significantly inhibited the expression of virulence genes (α and β toxins) of C. perfringens. Significant differences versus control group: *p < 0.05. (B) BSC10 and Lac16 cultures significantly inhibited C. perfringens growth. (C) Live Lac16 significantly protect C. elegans against C. perfringens infection. [file Image_1.tif]

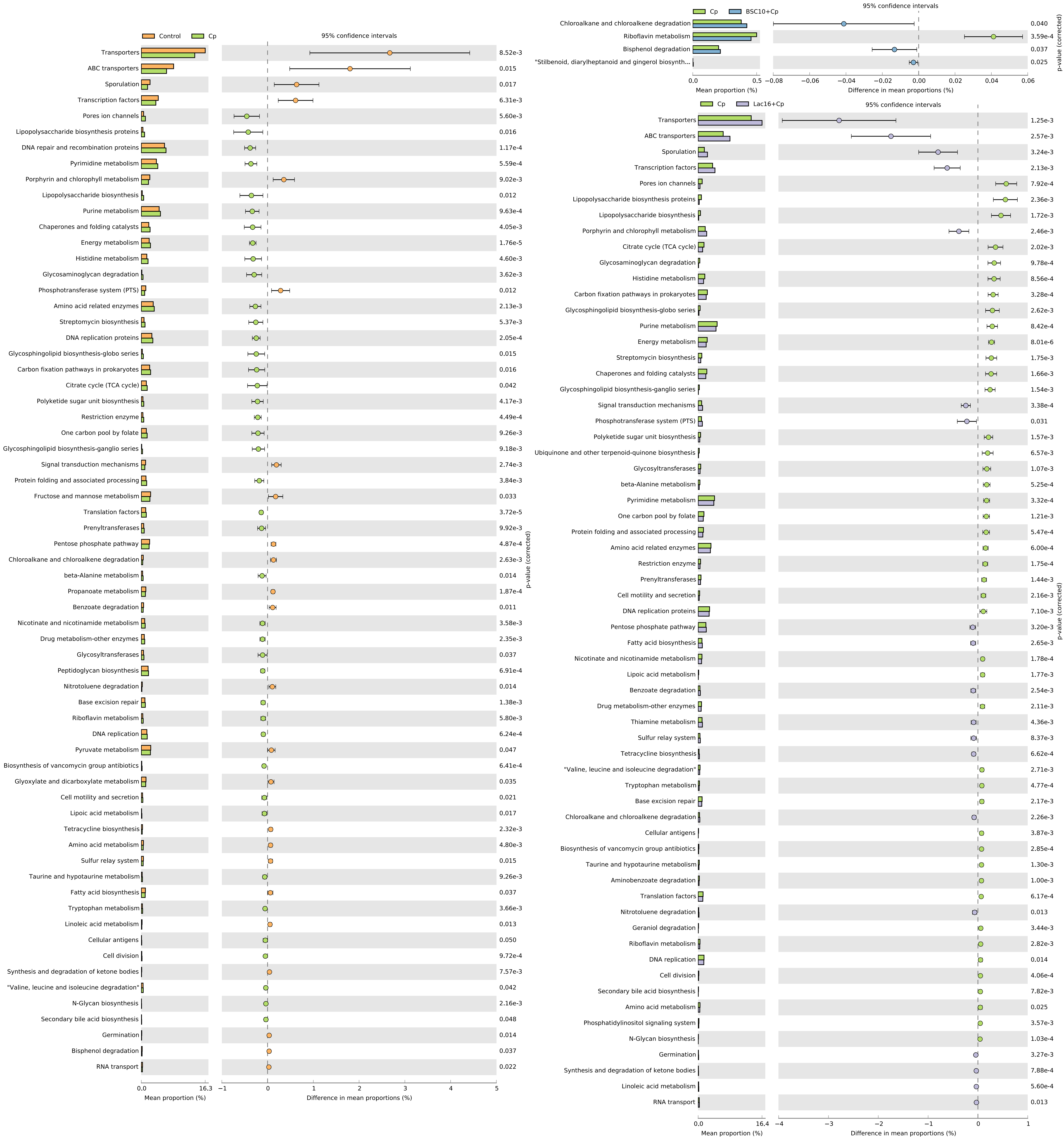

Supplement: Figure S2 — Comparison of predicted pathway abundances between the groups by statistical analysis of taxonomic and functional profiles (STAMP). (A) Control versus Cp; (A) Cp versus BSC10+Cp; (B) Cp versus Lac16+Cp. [file Image_2.pdf]

# Bacterial communities

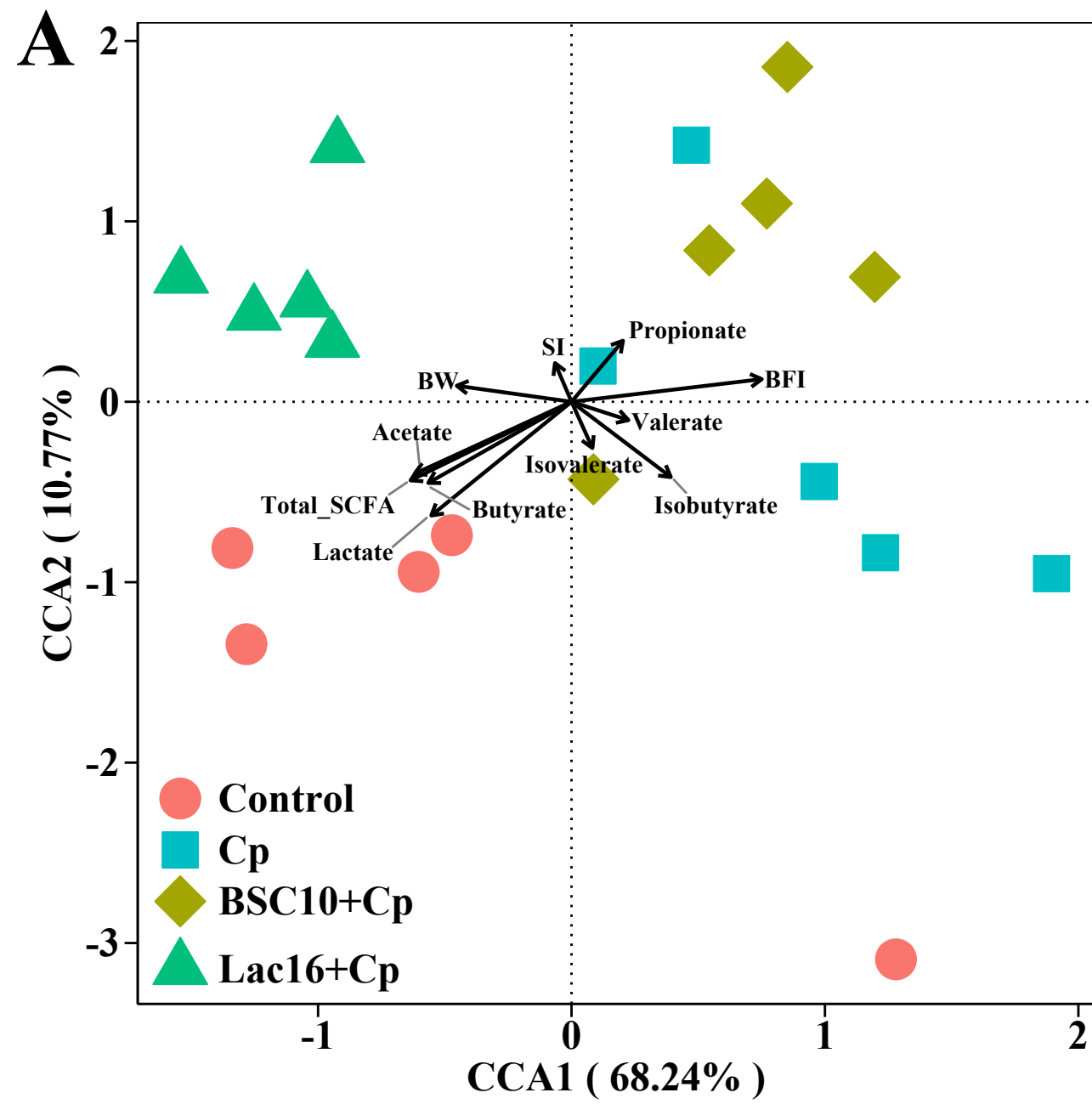

# Bacterial functions

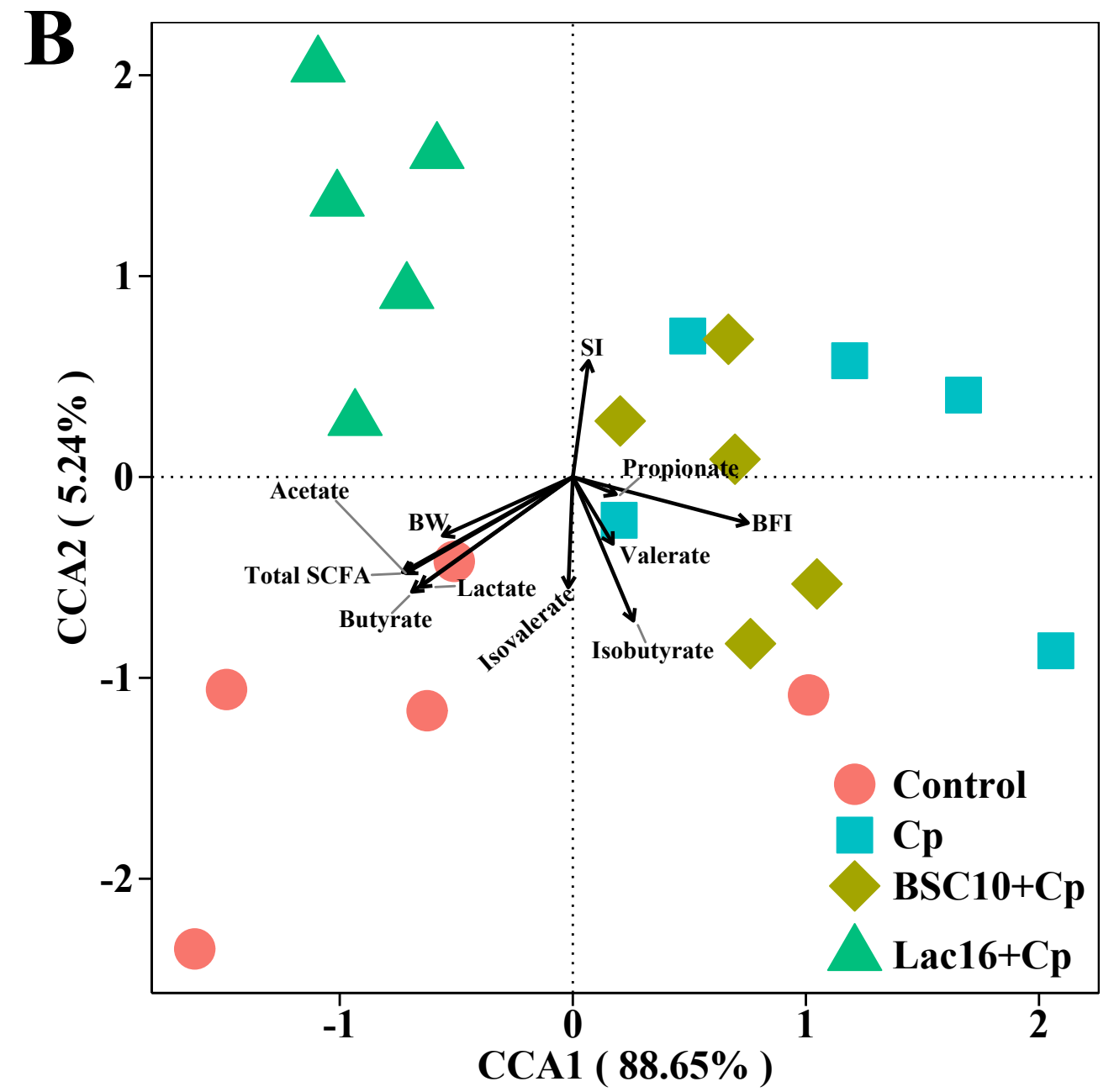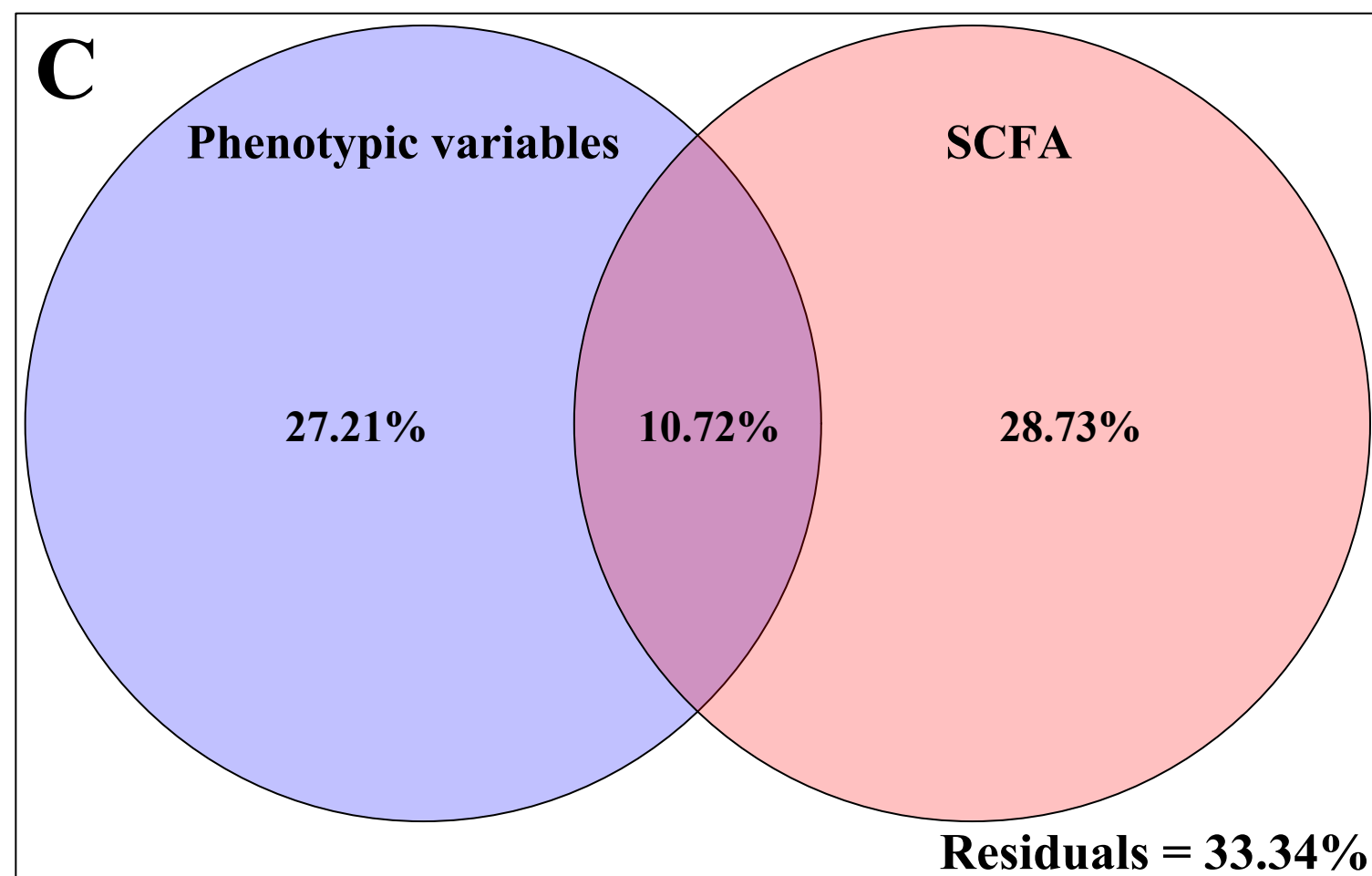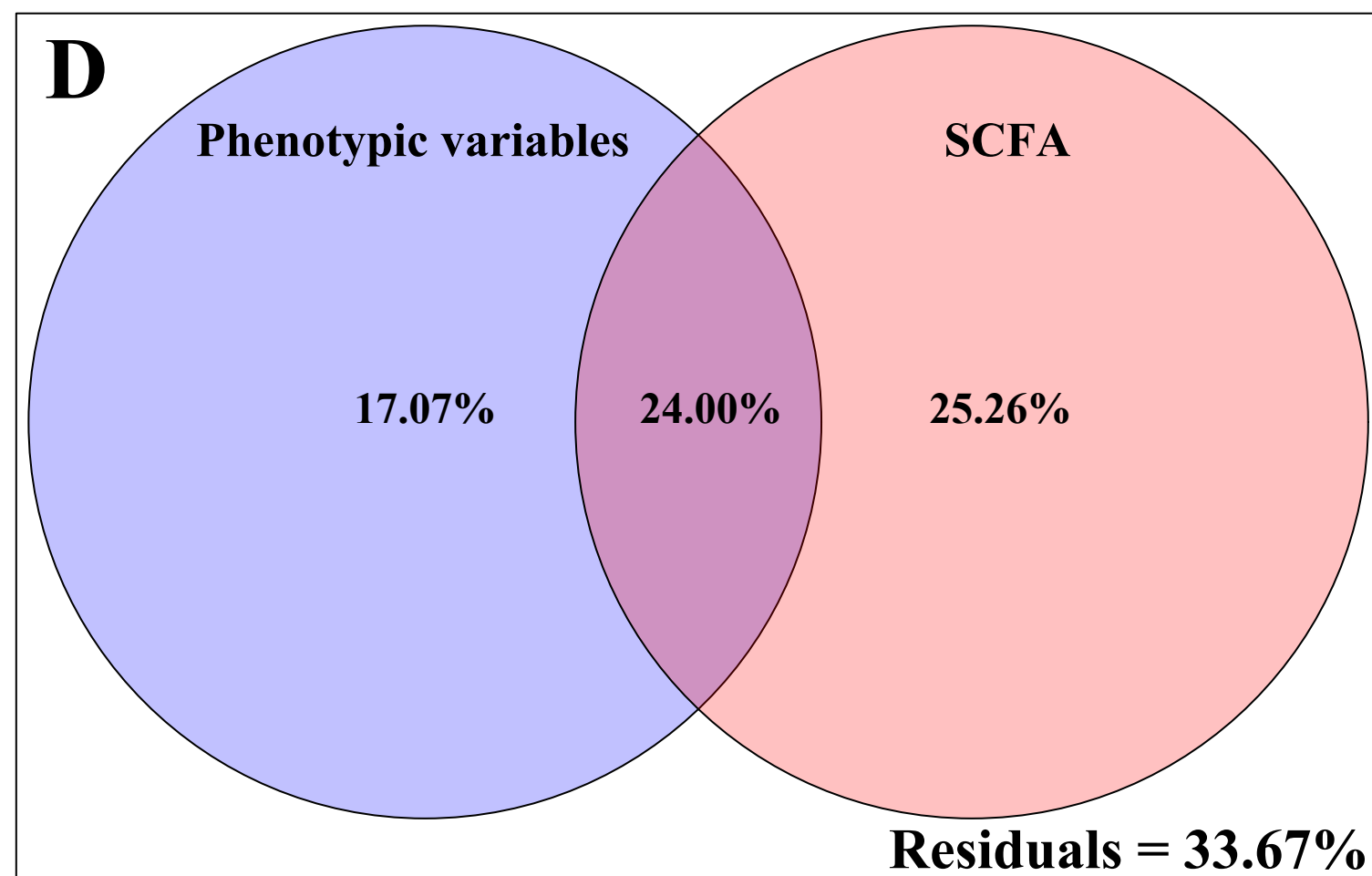

Supplement: Figure S3 — Canonical correspondence analysis and variation partitioning analysis of the phenotypic variables (including body weight, bursa of fabricius index and spleen index) and ileal SCFAs for the bacterial community (A,C) and microbial predicted pathway functions (B,D). [file Image_3.pdf]

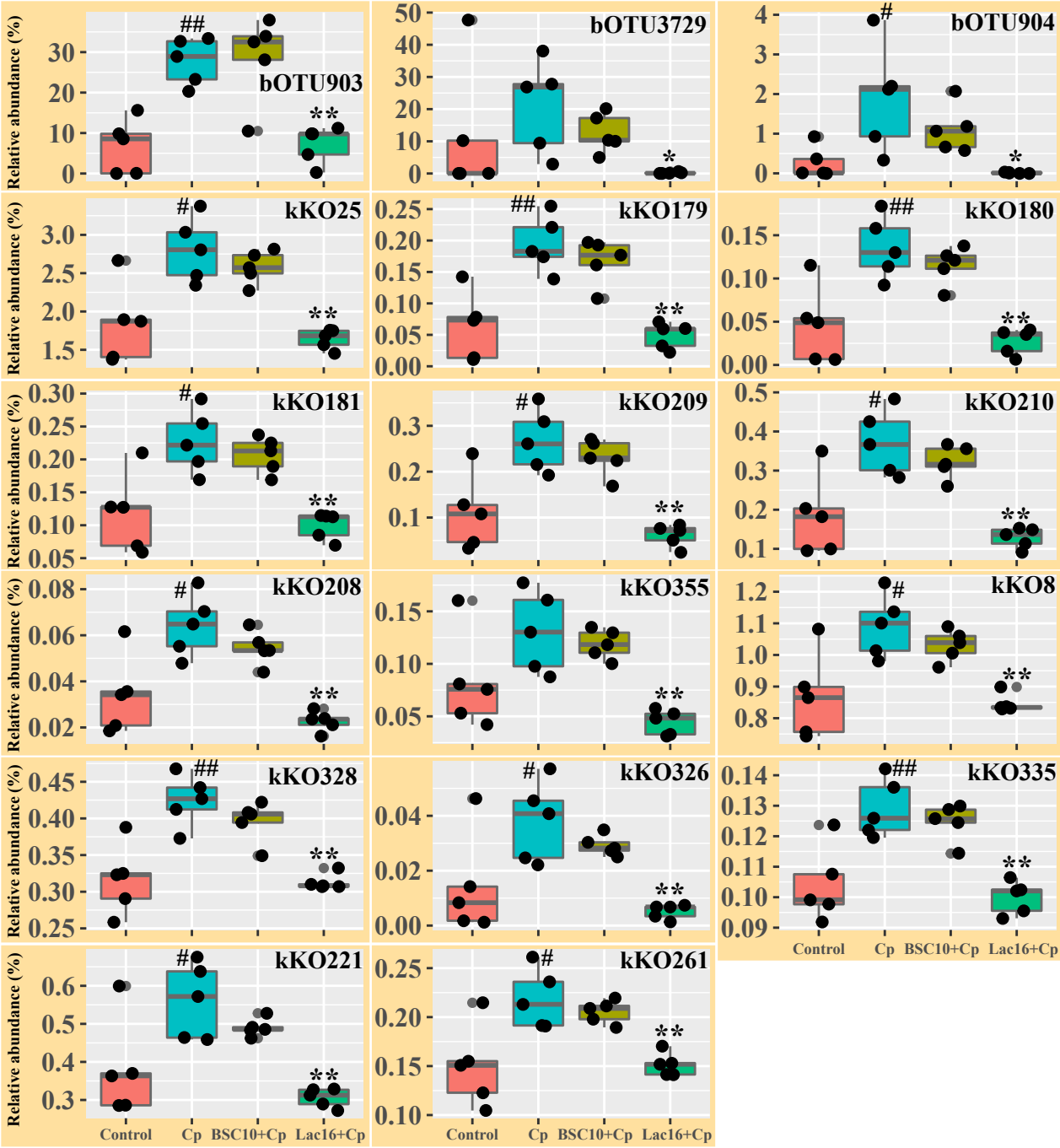

Supplement: Figure S4 — Relative abundances of the keystone species. Significant differences versus Control group: #p < 0.05; ##p < 0.01. Significant differences versus Cp group: *p < 0.05; **p < 0.01. The prefixes “b_” and “k_” represent the bacteria and KEGG. n = 5 samples. [file Image_4.pdf]
