# Supplementary material for: Dietary Supplementation With Lactobacillus plantarum Ameliorates Compromise of Growth Performance by Modulating Short-Chain Fatty Acids and Intestinal Dysbiosis in Broilers Under Clostridium perfringens Challenge
Source: Front Nutr. 2021 Oct 14;8:706148. doi: 10.3389/fnut.2021.706148 (PMC8551491; doi:10.3389/fnut.2021.706148)
Supplement: Supplementary file 5 [file Table_1.docx]

**Supplementary Material**

**TABLE S1. Composition and nutrient level of the basal diet (% as fed basis)**

| Ingredients | Content (%) | Nutrient levels^2^ | Content (%) |
| --- | --- | --- | --- |
| Corn | 60.00 | ME (MJ/kg) | 11.80 |
| Soy bean meal (48% CP) | 28.50 | Lysine | 1.21 |
| Fish meal | 2.00 | Methionine | 0.49 |
| Wheat middling | 4.50 | Methionine + cysteine | 0.65 |
| Salt | 0.30 | Threonine | 0.75 |
| Choline chloride (50%) | 0.15 | Arginine | 1.31 |
| CaHPO_4_ | 1.20 | CP | 21.01 |
| Limestone | 1.20 | Ca | 1.01 |
| Zeolite power | 1.20 | Available phosphorus | 0.47 |
| premix^1）^ | 1.00 | Total phosphorus | 0.69 |

1. Supplied per kilogram of diet: Vitamin A 25000 IU, Vitamin D 5000 IU, Vitamin E 12.5 IU, Vitamin K 1.25 mg, Vitamin B_1_ 1.0 mg, Vitamin B_2_ 8 mg, Vitamin B_12_ 15 μg, pantothenic acid 250 μg, pyridoxine 9.09 mg, biotin 22.50 mg, folic acid 1.67 mg, ZnSO_4_.H_2_O 180.93 mg, CuSO_4_.H_2_O 33.18 mg, FeSO_4_.H_2_O 247.75 mg, MnSO_4_.H_2_O 248.45 mg, Ca(IO_3_)_2_ 85.80 mg, Na_2_SeO_3_ 37.60 mg.
2. Calculated nutrient levels.

**TABLE S2** Microbial keystone OTUs identified in the rhizosphere soil microbial communities

| Phylum | Class | Order | Family | Genus | species | Node | Degree | tsNodes |
| --- | --- | --- | --- | --- | --- | --- | --- | --- |
| Bacteroidetes | Bacteroidia | Bacteroidales | Rikenellaceae |  |  | bOTU903 | 88 | Yes |
| Bacteroidetes | Bacteroidia | Bacteroidales | Bacteroidaceae | Bacteroides |  | bOTU3729 | 92 | Yes |
| Bacteroidetes | Bacteroidia | Bacteroidales | Bacteroidaceae | Bacteroides | unidentified | bOTU904 | 91 | Yes |

**TABLE S3** Microbial keystone KOs identified in the rhizosphere soil microbial communities

| Level 1 | Level 2 | Level 3 | Node | Degree | tsNodes |
| --- | --- | --- | --- | --- | --- |
| Metabolism | Glycan Biosynthesis and Metabolism |  | kKO25 | 91 | Yes |
| Metabolism | Glycan Biosynthesis and Metabolism | Glycosaminoglycan degradation | kKO179 | 88 | Yes |
| Metabolism | Glycan Biosynthesis and Metabolism | Glycosphingolipid biosynthesis-ganglio series | kKO180 | 89 | Yes |
| Metabolism | Glycan Biosynthesis and Metabolism | Glycosphingolipid biosynthesis-globo series | kKO181 | 88 | Yes |
| Metabolism | Glycan Biosynthesis and Metabolism | Lipopolysaccharide biosynthesis | kKO209 | 88 | Yes |
| Metabolism | Glycan Biosynthesis and Metabolism | Lipopolysaccharide biosynthesis proteins | kKO210 | 89 | Yes |
| Metabolism | Metabolism of Cofactors and Vitamins | Lipoic acid metabolism | kKO208 | 86 | Yes |
| Metabolism | Metabolism of Cofactors and Vitamins | Ubiquinone and other terpenoid-quinone biosynthesis | kKO355 | 88 | Yes |
| Metabolism | Biosynthesis of Other Secondary Metabolites |  | kKO8 | 87 | Yes |
| Metabolism | Biosynthesis of Other Secondary Metabolites | Streptomycin biosynthesis | kKO328 | 86 | Yes |
| Metabolism | Lipid Metabolism | Steroid hormone biosynthesis | kKO326 | 86 | Yes |
| Metabolism | Metabolism of Other Amino Acids | Taurine and hypotaurine metabolism | kKO335 | 86 | Yes |
| Unclassified | Cellular Processes and Signaling | Membrane and intracellular structural molecules | kKO221 | 88 | Yes |
| Cellular Processes | Transport and Catabolism | Peroxisome | kKO261 | 86 | Yes |

**FIGURE S1 Probiotics exerts anti-*C. perfringens* activity.** (A) Fermented supernatant of *P. polymyxa* (BSC10) and *L. plantarum* (Lac16) significantly inhibited the expression of virulence genes (α and β toxins) of *C. perfringens*. Significant differences versus control group: * *p* < 0.05. (B) BSC10 and Lac16 cultures significantly inhibited *C. perfringens* growth. (C) Live Lac16 significantly protect *C. elegans* against *C. perfringens* infection.

**FIGURE S2** Comparison of predicted pathway abundances between the groups by statistical analysis of taxonomic and functional profiles (**STAMP**). (A) Control versus Cp; (A) Cp versus BSC10+Cp; (B) Cp versus Lac16+Cp.

**FIGURE S3** Canonical correspondence analysis and variation partitioning analysis of the phenotypic variables (including body weight, bursa of fabricius index and spleen index) and ileal SCFAs for the bacterial community (A, C) and microbial predicted pathway functions (B, D).

**FIGURE S4** Relative abundances of the keystone species. Significant differences versus Control group: # *p* < 0.05; ## *p* < 0.01. Significant differences versus Cp group: * *p* < 0.05; ** *p* < 0.01. The prefixes “b_” and “k_” represent the bacteria and KEGG. n = 5 samples.
